# Preclinical model systems of ryanodine receptor 1-related myopathies and malignant hyperthermia: a comprehensive scoping review of works published 1990–2019

**DOI:** 10.1186/s13023-020-01384-x

**Published:** 2020-05-07

**Authors:** Tokunbor A. Lawal, Emily S. Wires, Nancy L. Terry, James J. Dowling, Joshua J. Todd

**Affiliations:** 1 National Institute of Nursing Research, National Institutes of Health, Bethesda, MD 20892 USA; 2 National Institute on Drug Abuse, National Institutes of Health, Baltimore, MD USA; 3 National Institutes of Health Library, National Institutes of Health, Bethesda, MD USA; 4 Program for Genetics and Genome Biology, Hospital for Sick Children, Toronto, Ontario Canada

**Keywords:** Ryanodine receptor, RYR1, Congenital myopathy, Central core disease, Preclinical, Mouse, Zebrafish, HEK-293, Porcine, Malignant hyperthermia

## Abstract

**Background:**

Pathogenic variations in the gene encoding the skeletal muscle ryanodine receptor (RyR1) are associated with malignant hyperthermia (MH) susceptibility, a life-threatening hypermetabolic condition and *RYR1*-related myopathies (*RYR1*-RM), a spectrum of rare neuromuscular disorders. In *RYR1*-RM, intracellular calcium dysregulation, post-translational modifications, and decreased protein expression lead to a heterogenous clinical presentation including proximal muscle weakness, contractures, scoliosis, respiratory insufficiency, and ophthalmoplegia. Preclinical model systems of *RYR1*-RM and MH have been developed to better understand underlying pathomechanisms and test potential therapeutics.

**Methods:**

We conducted a comprehensive scoping review of scientific literature pertaining to *RYR1*-RM and MH preclinical model systems in accordance with the PRISMA Scoping Reviews Checklist and the framework proposed by Arksey and O’Malley. Two major electronic databases (PubMed and EMBASE) were searched without language restriction for articles and abstracts published between January 1, 1990 and July 3, 2019.

**Results:**

Our search yielded 5049 publications from which 262 were included in this review. A majority of variants tested in *RYR1* preclinical models were localized to established MH/central core disease (MH/CCD) hot spots. A total of 250 unique *RYR1* variations were reported in human/rodent/porcine models with 95% being missense substitutions. The most frequently reported *RYR1* variant was R614C/R615C (human/porcine total *n* = 39), followed by Y523S/Y524S (rabbit/mouse total *n* = 30), I4898T/I4897T/I4895T (human/rabbit/mouse total *n* = 20), and R163C/R165C (human/mouse total *n* = 18). The dyspedic mouse was utilized by 47% of publications in the rodent category and its RyR1-null (1B5) myotubes were transfected in 23% of publications in the cellular model category. In studies of transfected HEK-293 cells, 57% of *RYR1* variations affected the RyR1 channel and activation core domain. A total of 15 *RYR1* mutant mouse strains were identified of which ten were heterozygous, three were compound heterozygous, and a further two were knockout. Porcine, avian, zebrafish, *C. elegans*, canine, equine, and drosophila model systems were also reported.

**Conclusions:**

Over the past 30 years, there were 262 publications on MH and *RYR1*-RM preclinical model systems featuring more than 200 unique *RYR1* variations tested in a broad range of species. Findings from these studies have set the foundation for therapeutic development for MH and *RYR1*-RM.

## Introduction

Ryanodine receptor 1-related myopathies (*RYR1*-RM) are a diverse spectrum of rare monogenic neuromuscular disorders that manifest from variations in the *RYR1* gene [1, 2]. In total, > 700 *RYR1* variations have been identified; many of which are private to an individual case or family [3]. *RYR1* exhibits little functional variation (per a recently developed bioinformatic residual variance intolerance [RVIS] scoring system: − 8.29 [0.01%]) [4] and encodes a 2.2 megadalton homotetrameric calcium ion channel (RyR1) that is localized to the sarcoplasmic reticulum (SR) membrane in skeletal muscle [5]. The physical connection between the RyR1 cytosolic shell and dihydropyridine receptor (DHPR) enables a coordinated release of SR calcium to the muscle cell cytosol, a process that facilitates excitation-contraction coupling in response to depolarization of the transverse-tubule membrane [6, 7]. ER/SR calcium concentration is an estimated 1000–10,000 times greater than cytosolic calcium concentration, and maintenance of this steep gradient is imperative to the health of the cell [8, 9]. Preclinical studies have identified intracellular calcium dysregulation as the central pathomechanism resulting from *RYR1* variations characterized by SR calcium leak or excitation-contraction uncoupling [10]. In addition, the presence of truncation variations often reported in compound heterozygous cases can lead to decreased RyR1 expression [11, 12]. Owing to > 100 cysteine residues per subunit, RyR1 are susceptible to post-translational modifications, which in the case of mutant channels, further exacerbate intracellular calcium dysregulation though a previously reported feed-forward mechanism [13, 14]. For example, an elevated level of S-nitrosylated cysteines greatly increases channel activity, thus perpetuating calcium release. *RYR1*-RM pathomechanisms have been reviewed in detail elsewhere [10].

*RYR1*-RM can be inherited in a dominant or recessive manner and are slowly progressive with clinical manifestations including proximal muscle and facial weakness, joint contractures, scoliosis, ophthalmoplegia, and respiratory muscle weakness [15]. Although presentation often occurs at birth or in early childhood, adult-onset cases have also been reported [16, 17]. Affected individuals are considered at risk of malignant hyperthermia (MH) susceptibility. Genetic predisposition to MH can result in a potentially fatal hypermetabolic response and skeletal muscle rigidity upon exposure to triggers such as volatile anesthetics, exercise in the heat, and influenza [18, 19]. In addition to myopathy, other clinical phenotypes attributed to *RYR1* variations include rhabdomyolysis-myalgia syndrome and intermittent periodic paralysis [20, 21]. Historically, *RYR1*-RM were sub-categorized based on skeletal muscle histopathology. This yielded subtypes such as central core disease, multiminicore disease, centronuclear myopathy, and congenital fiber-type disproportion [22]. Despite being the most frequently reported non-dystrophic neuromuscular disorder [23], there is currently no approved treatment for *RYR1*-RM.

A decade after the first report of central core disease in humans [24], Hall and colleagues observed a fatal hypermetabolic response to suxamethonium in pigs [25]. This was the first MH animal model system whose phenotype, also referred to as porcine stress syndrome, was later attributed to the R615C variation in *RYR1* [26, 27]*.* Since this landmark discovery, technological and scientific advances have led to the development of preclinical model systems that can be grouped into cell culture and animal categories, each with their own advantages and limitations [28–32].

### Objective

The objective of this scoping review was to comprehensively review the scientific literature for MH and *RYR1*-RM preclinical model systems, thus generating a resource to guide future research.

## Methods

The PRISMA extension for Scoping Reviews (PRISMA-ScR) Checklist and the framework proposed by Arksey and O’Malley [33] were used to guide this scoping review. The overarching research question was: what preclinical model systems have been reported for MH and *RYR1*-RM?

### Identifying relevant studies

Two major electronic databases (PubMed and EMBASE) were searched without language restriction for articles and abstracts published between January 1, 1990 and July 3, 2019. The search strategy comprised the following a priori search terms present in the title or abstract using Boolean operators and MeSH terms: RYR-1 OR RYR1 OR RyR1s OR ryanodine receptor calcium release channel OR “ryanodine receptor 1” AND malignant hyperthermia OR “malignant hyperthermia” OR malignant hyperpyrexia OR anesthesia hyperthermia OR Muscular diseases OR muscular diseases OR myopathies OR myopathy OR muscle OR muscular OR muscle contraction OR muscle contraction OR smooth muscle OR cardiac muscle OR skeletal muscle OR muscle fiber OR myofibril. The full search strategy is provided in Additional file 1.

### Study selection

Following removal of duplicates, titles and abstracts of all publications were reviewed independently by two of the authors and marked for inclusion if they discussed a MH or *RYR1*-RM preclinical model system. Publications were marked for exclusion if they were (1) not gene or isoform of interest (e.g. *CACNA1S*-related MH), (2) clinical report, (3) structural biology, (4) wild-type models and methods, (5) cardiac or smooth muscle, (6) review articles, or (7) categorized as miscellaneous. All publications were discussed with a third author who adjudicated when there was discordance between the first two authors over whether publication should be included or excluded.

### Charting data and reporting the results

The following data were extracted from full text publications selected for inclusion in the review: first author, year of publication, title of the publication, variation(s) of the preclinical model system(s), and conclusions of the publication on the disease model system(s). Data were tabulated according to type of preclinical model system. Categories included transfected human embryonic kidney cell (HEK)-293 cells, transfected *RYR1*-null (dyspedic) myotubes, immortalized B-lymphocytes, primary cell culture, porcine model systems, and rodent model systems. Data on all other preclinical model systems, including zebrafish, avian, *C. elegans*, and drosophila, were combined and tabulated separately. Two authors reviewed data extracted for each article. To identify gaps in the literature where no preclinical model system had been reported for a specific *RYR1* protein-coding region, the number of publications per *RYR1* exon was mapped against established MH/CCD hotspot regions and sequence of the RyR1 protein structure. The composition of included and excluded publications was also summarized.

## Results

### Study characteristics

**Fig. 1:**
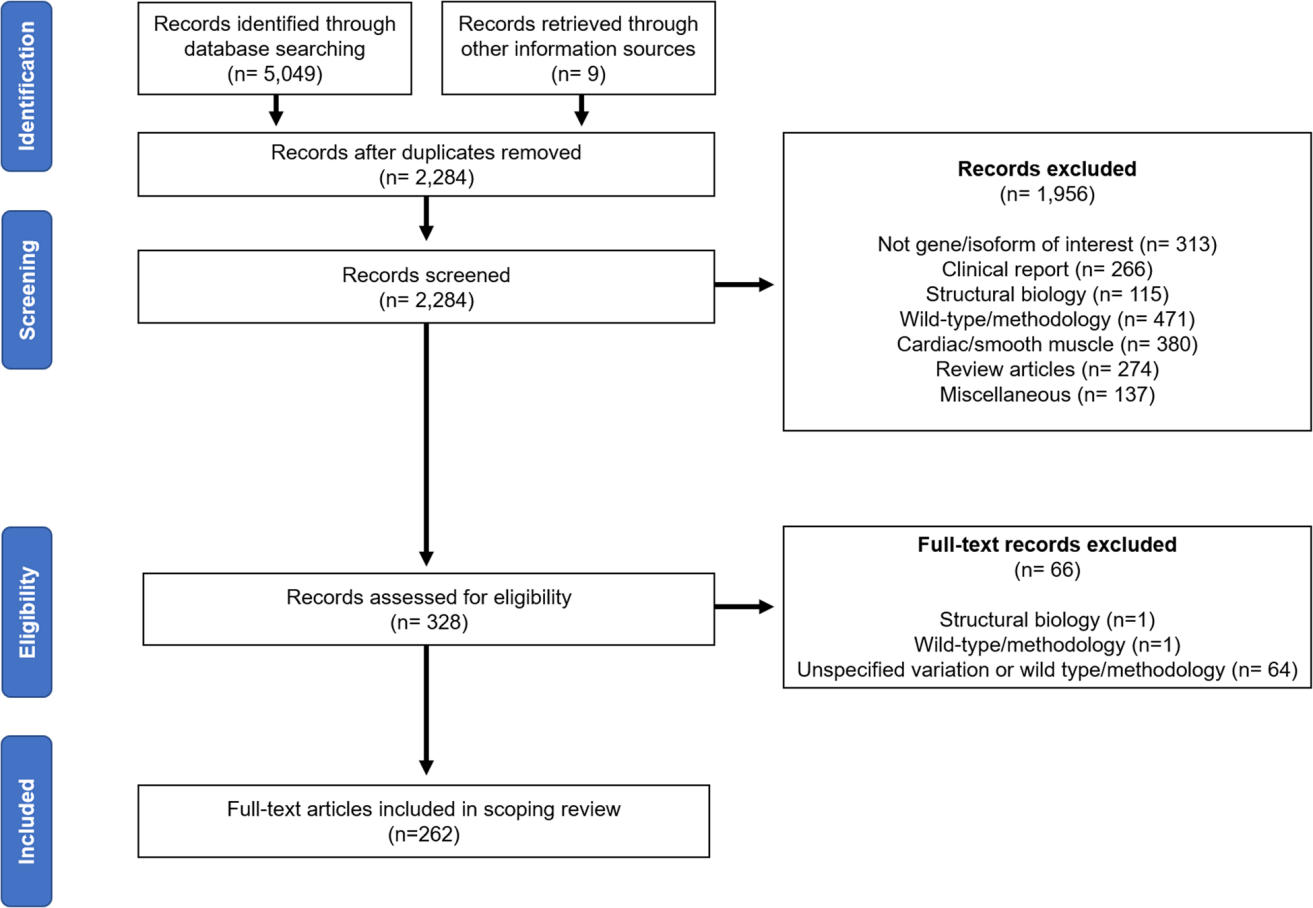
PRISMA diagram summarizing the article selection workflow

The search strategy utilized in this study yielded 5049 research publications between January 1, 1990 and July 3, 2019. Nine additional publications were retrieved through other information sources. Following removal of 2814 duplicates, 2284 abstracts were screened for inclusion. A total of 1956 publications were excluded at this point, leaving 328 for full text review. During full text review, 66 additional publications were excluded leaving 262 publications for inclusion in this review. An overview of this process is provided in Fig. 1.

**Fig. 2:**
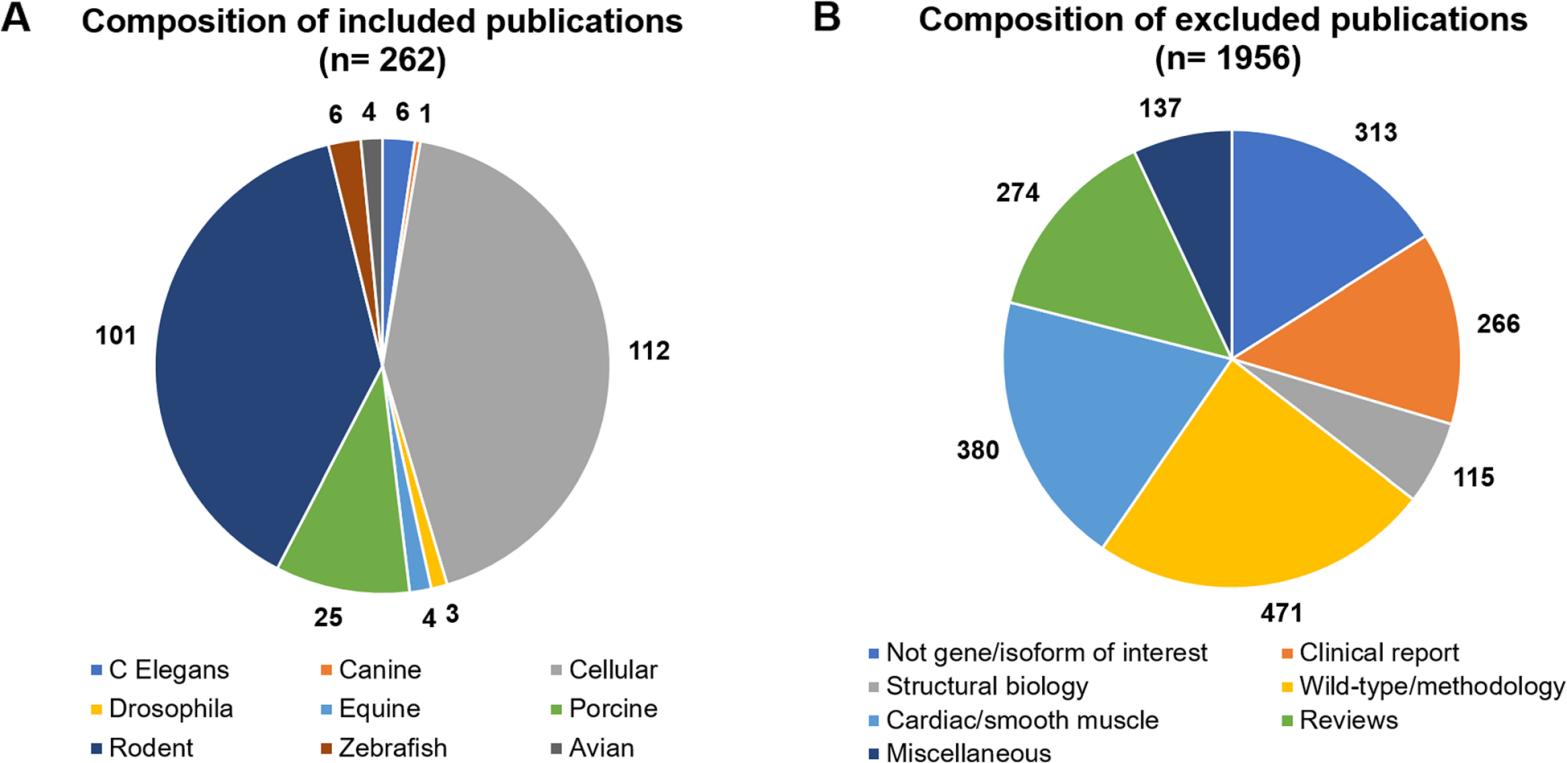
**a**-**b** Composition of included and excluded publications

The majority of publications that met inclusion criteria for this review focused on *RYR1* cellular and rodent model systems (43 and 39%, and respectively), Fig. 2a. Wild-type/methods publications formed the largest group of those excluded (24%), followed by those focused on cardiac/smooth muscle (19%), not isoform/gene of interest (16%), and clinical reports (13%), Fig. 2b.

**Fig. 3:**
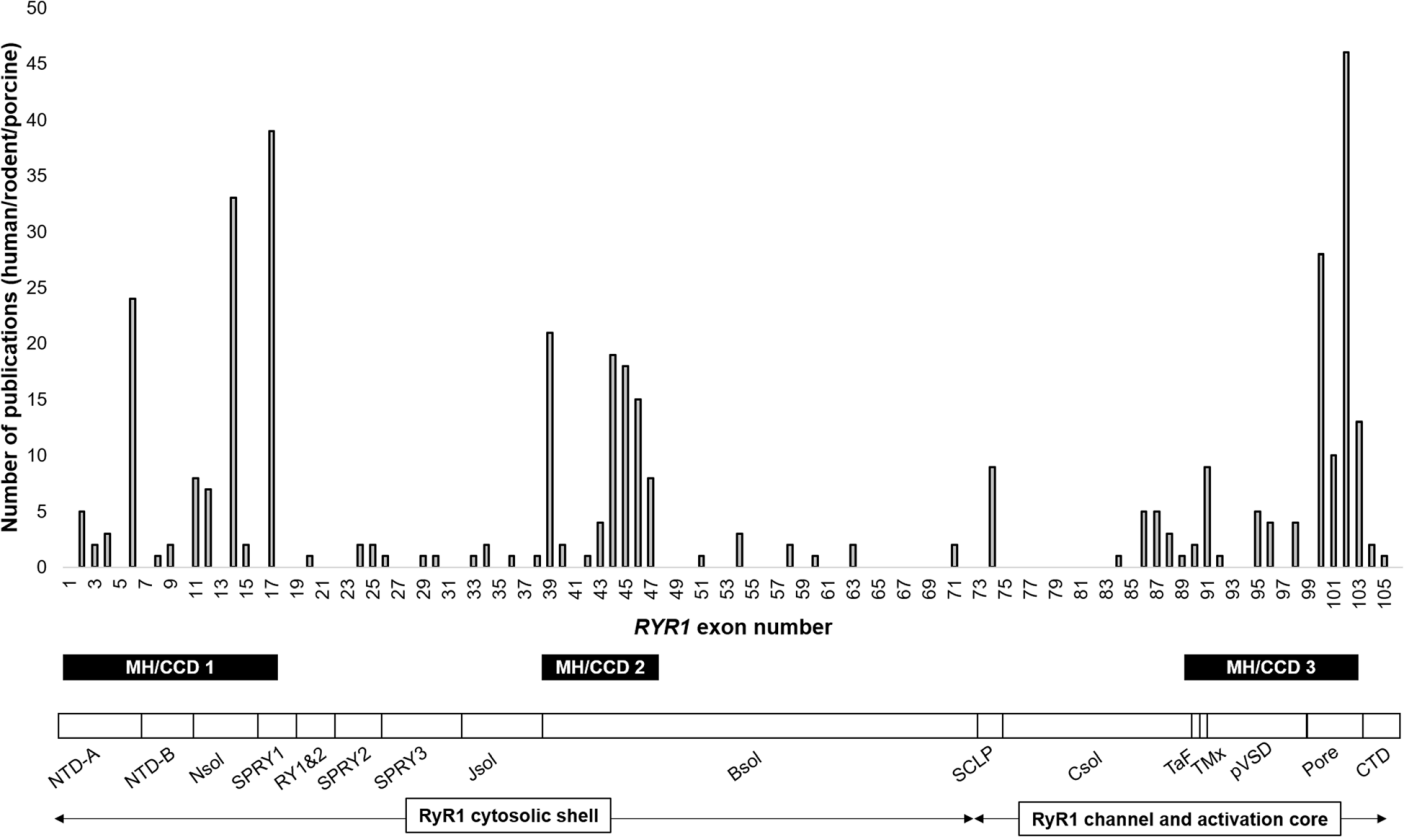
Number of publications per *RYR1* exon aligned with corresponding MH/CCD hotspots and RyR1 structural regions

The highest frequencies of variations reported in *RYR1* preclinical model systems were localized to established MH/central core disease (MH/CCD) hot spots 1, 2, and 3 located between exons 1–17, 39–46, and 90–103, respectively, Fig. 3. At least one *RYR1* preclinical model system was reported for every RyR1 structural region.

**Fig. 4:**
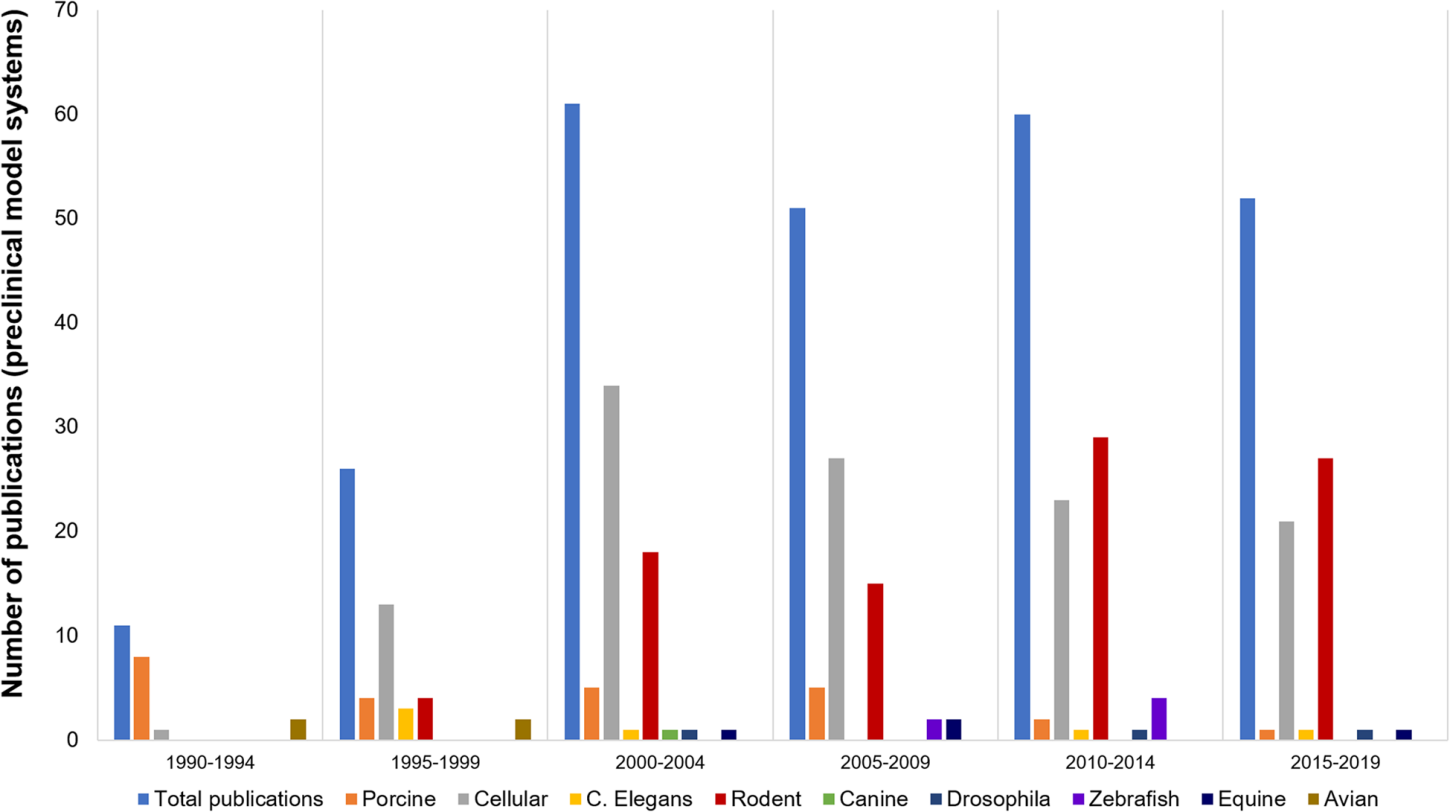
Total number of publications over time and type of *RYR1* preclinical model system reported

A total of 250 unique *RYR1* variations were reported in human/mouse/porcine model systems with 95% being missense substitutions. The most frequently reported *RYR1* variations reported across species were R614C/R615C (human/porcine total *n* = 39), Y523S/Y524S (rabbit/mouse total *n* = 30), I4898T/I4897T/I4895T (human/rabbit/mouse total *n* = 20), and R163C/R165C (human/mouse total *n* = 18). The dyspedic mouse was the most frequently reported mouse model system comprising 47% of publications in this category. The predominant type of *RYR1* preclinical model system used has varied over time. From 1990 to 1994, the R615C porcine model system was most frequently reported. Cellular model systems were then most frequently reported until 2010, after which this transitioned to rodent model systems including RyR1-null (dyspedic) and Y524S, R163C, and I4895T mutant mice, Fig. 4.

### Cellular model systems

#### Expression of recombinant RYR1 in heterologous cells

A total of 49 publications reported transfecting mutant *RYR1* cDNA into HEK-293 cells, which lack native RyR1 channels, making this the most frequently utilized cellular model, Table 1. These 49 publications reported on 161 unique *RYR1* variations of which 153 were missense substitutions, six were deletions, one was a frameshift variant resulting in a truncation, and one was a deletion-insertion resulting in a truncation. Of these unique variations, 57% affected the RyR1 channel and activation core domain. In the 49 publications reporting on mutant HEK-293 cells, 13 variations were evaluated and/or functionally characterized (at least three times) (C36R [47, 81, 83], R164C [43, 47, 81, 83], G249R [47, 81, 83], G342R [39, 47, 81, 83], Y523S [43, 47, 70, 81, 83], R615C [47, 70, 81, 83], R2163C [45, 81, 83], R2163H [45, 81, 83], R2435H [39, 43, 45], R2458C [45, 81, 83], R2458H [45, 81, 83], R2508C [45, 52, 61], R2508H [45, 48, 52]). A majority (13/14) of these well-characterized missense substitutions affected the RyR1 cytosolic shell domain. In four publications, multiple variants were introduced to HEK-293 cells to evaluate their impact alone and in combination on RyR1 structural conformation and calcium homeostasis [35–37, 67]. Two additional publications reported on monkey-derived CV-1 in Origin with SV40 (COS)-7 cells that were transfected with mutant *RYR1* cDNA [84, 85].

**Table 1:** Cellular *RYR1* model systems: Human embryonic kidney (HEK-293) cells

Author/Year	***RYR1*** variant(s)	Title	Conclusions
HEK-293			
Chirasani VR, et al. [34] 2019	Q3970K, Q3970E	A central core disease mutation in the Ca^2+^ binding site of skeletal muscle ryanodine receptor impairs single channel regulation	RyR1-Q3970K is likely a CCD-associated loss-of-function channel that conducts Ca^2+^
Xu L, et al. [35] 2018	G4934D, G4934K, G4941D, G4941K, G4941M, D4938N, D4945N	G4941K substitution in the pore-lining S6 helix of the skeletal muscle ryanodine receptor increases RyR1 sensitivity to cytosolic and luminal Ca^2+^	Luminal Ca^2+^ accesses Ca^2+^ activation sites as they pass through the pore rather than traveling to openings that lie outside the pore
Xu L, et al. [36] 2018	E3893Q, E3893V, E3967Q, E3967V, T5001A	Ca^2+^ − mediated activation of the skeletal-muscle ryanodine receptor ion channel	Removal of negative charges in a RyR1 Ca^2+^ binding site impairs activation of RyR1 by physiological concentrations of Ca^2+^, and suggests loss of binding to or reduced Ca^2+^ affinity of the site
Xu L, et al. [37] 2008	G4898E, G4898R, ΔV4926, ΔI4927, R110W, L486V	Single channel properties of heterotetrameric mutant RyR1 ion channels linked to core myopathies	Homozygous RyR1 mutations associated with core myopathies abolish or greatly reduce sarcoplasmic reticulum Ca^2+^ release during excitation-contraction coupling
Schiemann AH, et al. [38] 2018	D2431Y	A genetic mystery in malignant hyperthermia ‘solved’?	The D2431Y variant is pathogenic for MH and should be added to the European Malignant Hyperthermia Group (EMHG) list of diagnostic mutations
Murayama T, et al. [39] 2018	G342R, R2435H, L4824P	Efficient High-Throughput Screening by Endoplasmic Reticulum Ca^2+^ Measurement to Identify Inhibitors of Ryanodine Receptor Ca^2+^ − Release Channels	In the current high throughput screening of 1535 compounds, we identified four RyR1 inhibitors
Kondo T, et al. [40] 2018	T84M	Genetic and functional analysis of the *RYR1* mutation pThr84Met revealed a susceptibility to malignant hyperthermia	Functional analysis of T84M demonstrated higher responsivity to caffeine and 4CmC
Parker R, et al. [41] 2017	M4640I, V4849I, F4857S, D4918N	Functional Characterization of C-terminal Ryanodine Receptor 1 Variants Associated with Central Core Disease or Malignant Hyperthermia	The V4849I variant should be considered a risk factor for malignant hyperthermia, while the F4857S and D4918N variants should be classified as pathogenic for CCD
Merritt A, et al. [42] 2017	R2336H, R2355W, E3104K, G3990V, V4849I, D3986E	Assessing the pathogenicity of *RYR1* variants in malignant hyperthermia	Functional analyses in HEK293 cells provided evidence to support the use of R2336H, R2355W, E3104K, pG3990V and V4849I for diagnostic purposes but not D3986E
Chen W, et al. [43] 2017	R164C, Y523S, R2136H, R2435H, Y4796C	Reduced threshold for store overload-induced Ca^2+^ release is a common defect of RyR1 mutations associated with malignant hyperthermia and central core disease	All mutations reduced the threshold for SOICR
Stephens J et al. [44] 2016	ΔE2348, T214M	Functional analysis of *RYR1* variants linked to malignant hyperthermia	ΔE2348 could be added to the list of diagnostic mutations for susceptibility to malignant hyperthermia T214M, does not appear to significantly alter sensitivity to agonist in the same system
Murayama T, et al. [45] 2016	R2163C, R2163H, V2168M, T2206M, A2350T, G2375A, G2434R, R2435H, R2454C, R2454H, R2458C, R2458H, R2508C, R2508H	Genotype–Phenotype Correlations of Malignant Hyperthermia and Central Core Disease Mutations in the Central Region of the *RYR1* Channel	In live-cell Ca^2+^ imaging, the mutant channels exhibited an enhanced sensitivity to caffeine, a reduced endoplasmic reticulum Ca^2+^ content, and an increased resting cytoplasmic Ca^2+^ level
Gomez AC, et al. [46] 2016	F4732D, G4733E, R4736W, R4736Q, T4825I, H4832Y, T4082M, S4113L, N4120Y	Malignant hyperthermia-associated mutations in the S2-S3 cytoplasmic loop of type 1 ryanodine receptor calcium channel impair calcium-dependent inactivation	Nine RyR1 mutants associated with skeletal muscle diseases were differently regulated by Ca^2+^ and Mg^2+^
Murayama T, et al. [47] 2015	C36R, R164C, R164L, G249R, G342R, R402C, R402H, Y523C, Y523S, R615C, R615L	Divergent Activity Profiles of Type 1 Ryanodine Receptor Channels Carrying Malignant Hyperthermia and Central Core Disease Mutations in the Amino-Terminal Region	The mutations increased the gain and the sensitivity to activating Ca^2+^ in a site-specific manner. Gain was consistently higher in both MH and MH/CCD mutations
Miyoshi H, et al. [48] 2015	R2508H, R2508G, R2508S, R2508K	Several Ryanodine Receptor Type 1 Gene Mutations of pArg2508 Are Potential Sources of Malignant Hyperthermia	Cells transfected with each of the 4 mutants, R2508H, R2508G, R2508S, or R2508K, were more sensitive to caffeine and 4CmC than wild-type cells
Mei Y, et al. [49] 2015	G4934A, G4934V, G4941V, G4941A, G4941I	Channel Gating Dependence on Pore Lining Helix Glycine Residues in Skeletal Muscle Ryanodine Receptor	Both glycines are important for RyR1 channel function by providing flexibility and minimizing amino acid clashes
Shirvanyants D, et al. [50] 2014	M4887G, M4887A, M4887V, V4891A, I4897Y	Pore dynamics and conductance of RyR1 transmembrane domain	Loss of these interactions in the case of polar substitution I4897T results in destabilization of the selectivity filter, a possible cause of the CCD-specific reduced Ca^2+^ conductance
Roesl C, et al. [51] 2014	R2452W	Functional characterisation of the R2452W ryanodine receptor variant associated with malignant hyperthermia susceptibility	R2452W results in a hypersensitive ryanodine receptor 1 and is likely to be causative of MH
Miyoshi H, et al. [52] 2014	R2508C, R2508H, R2508K, R2508S	Two different variants of p.2508 in Japanese malignant hyperthermia patients causing hypersensitivity of ryanodine receptor 1	All alterations in the p.2508 portion of RyR1 play important roles in the pathogenesis of MH
Sato K, et al. [53] 2013	R44C, R163C, R401C, R533C, R533H, H4833Y	Skeletal muscle ryanodine receptor mutations associated with malignant hyperthermia showed enhanced intensity and sensitivity to triggering drugs when expressed in human embryonic kidney cells	These six mutations cause functional abnormality of the calcium channel, leading to higher sensitivity to a specific agonist
Kraeva N, et al. [54] 2013	M4640R, L4647P, F4808L, D4918N, F4941C	Novel excitation-contraction uncoupled *RYR1* mutations in patients with central core disease	Homotetrameric RyR1 mutants harbouring L4646P, F4807P, D4917N and R4892Q mutations abolished caffeine-induced Ca^2+^ release
Merritt A, et al. [55] 2012	D1056H	Functional analysis of the pD1056H *RYR1* variant associated with malignant hyperthermia and exertional heat stroke	Cells expressing D1056H exhibited a trend for greater calcium release and increased sensitivity than wild-type at low doses of caffeine
Murayama T, et al. [56] 2011	T4825A, T4825I, I4826A, L4827A, S4828A, S4829A	Role of amino-terminal half of the S4-S5 linker in type 1 ryanodine receptor (RyR1) channel gating	Four mutants had reduced CICR activity without changing Ca^2+^ sensitivity, whereas the L4827A mutant formed a constitutive active channel T4825I, a disease-associated mutation for malignant hyperthermia, exhibited enhanced CICR activity
Haraki T, et al. [57] 2011	A4894T, A4894P, A4894S, A4894G	Mutated p.4894 RyR1 function related to malignant hyperthermia and congenital neuromuscular disease with uniform type 1 fiber (CNMDU1)	The hypersensitive A4894T-RyR1 is associated with MH and the poorly functional A4894P-RyR1 with CNMDU1
Zhou H, et al. [58] 2010	R2939K	Multi-minicore disease and atypical periodic paralysis associated with novel mutations in the skeletal muscle ryanodine receptor (RYR1) gene	The R2435K mutation did not affect two characteristic functional properties of RyR1, as both Ca^2+^ dependence and activation by caffeine were not altered
Sato K, et al. [59] 2010	R163C, G248R, T4826I, H4833Y, I4898T, G4899R	Functional studies of *RYR1* mutations in the skeletal muscle ryanodine receptor using human *RYR1* complementary DNA	MH mutations showed a higher response, whereas CCD mutants (I4898T and G4899R) did not respond to 4-Cm C
Merritt A, et al. [60] 2010	G3990V	Functional analysis of the pGly3990Val *RYR1* variant using a human cDNA clone in HEK293 cells	A statistically significant increase in Ca^2+^ release was observed in G3990V mutants at each caffeine concentration that elicited a response
Migita T, et al. [61] 2009	R2508C	Functional analysis of ryanodine receptor type 1 pR2508C mutation in exon 47	The transfected *RYR1* mutant was more sensitive to caffeine and 4CmC than wildtype *RYR1*
Migita T, et al. [62] 2009	R2508C, A4894T	Do Ca^2+^ channel blockers improve malignant hyperthermia crisis?	The dantrolene-induced decline effect of Ca^2+^ of skeletal muscle was not disappeared in the presence of Ca^2+^ blockers. In MH crisis, we do not recommend to administer Ca^2+^ blockers because of its potent effect to increase Ca^2+^
Ghassemi F, et al. [63] 2009	R2435L	A recessive ryanodine receptor 1 mutation in a CCD patient increases channel activity	R2435L does not affect resting Ca^2+^, or sensitivity of RyR1 to pharmacological activators Instead it reduces the release of Ca^2+^ from intracellular stores induced by pharmacological activators as well as by KCl via the voltage sensing dihydropyridine receptor
Jiang D, et al. [64] 2008	R615C	Reduced threshold for luminal Ca^2+^ activation of RyR1 underlies a causal mechanism of porcine malignant hyperthermia	R615C confers MH susceptibility by reducing the threshold for luminal Ca^2+^ activation and SOICR
Rossi D, et al. [65] 2007	R4836fsX4838	A truncation in the *RYR1* gene associated with central core lesions in skeletal muscle fibres	Subtle changes in Ca^2+^ release of human heteromeric RyR1/RyR1R4837fsX4839 channels, probably due to the reduced stability/assembly of these channels, may predispose individuals to MHS
Lyfenko AD, et al. [66] 2007	R4214_F4216del, V4927_I4928del	Two central core disease (CCD) deletions in the C-terminal region of *RYR1* alter muscle excitation-contraction (EC) coupling by distinct mechanisms	Single channel data indicate that the ΔRQF mutation increases Ca^2+^ responsiveness without altering K^+^ conductance and ion selectivity for Ca^2+^ compared to K^+^. In contrast, the ΔVI deletion abolished Ca^2+^ responsiveness, Ca^2+^ permeation, and significantly reduced K^+^ conductance demonstrating that the ΔVI mutation introduced major alterations to the channel pore
Zhou H, et al. [67] 2006	S71Y, R110W, L486V, A1578T, S2060C, N2283H	Characterization of recessive *RYR1* mutations in core myopathies	Recombinant channels with N2283H substitution showed an increased activity, whereas recombinant channels with S71Y + N2283H substitution lost activity upon isolation
Xu L, et al. [68] 2006	D4938N, D4945N, D4953N, E4942Q, E4948Q, E4952Q, E4955Q	Two rings of negative charges in the cytosolic vestibule of type-1 ryanodine receptor modulate ion fluxes	D4938N and D4945N exhibited an attenuated block by neomycin to a greater extent from the cytosolic than lumenal side. By comparison, charge neutralization of lumenal loop residues (D4899Q, E4900N) eliminated the block from the lumenal but not the cytosolic side
Wang Y, et al. [69] 2005	D4899Q, E4900N	Probing the role of negatively charged amino acid residues in ion permeation of skeletal muscle ryanodine receptor	the negatively charged carboxyl oxygens of D4899 and E4900 side chains are major determinants of RyR ion conductance and selectivity
Brini M, et al. 2005 [70]	R615C, Y523S, I4898T	Ca^2+^ signaling in HEK-293 and skeletal muscle cells expressing recombinant ryanodine receptors harboring malignant hyperthermia and central core disease mutations	I4898T RyR1 channels produced cytosolic Ca^2+^ values which were similar to those observed for WT RyR1 channels R615C augmented the amplitude of the cytosolic and mitochondrial Ca^2+^ transients following cell stimulation By contrast, the mitochondrial Ca^2+^ transients were reduced in cells expressing Y523S
Du GG, et al. [71] 2004	R4892W, I4897T, G4898E	Central core disease mutations R4892W, I4897T and G4898E in the ryanodine receptor isoform 1 reduce the Ca^2+^ sensitivity and amplitude of Ca^2+^-dependent Ca^2+^ release	Ca^2+^ sensitivity is one of the serious defects in these three excitation-contraction uncoupling CCD mutations
Zozato F, et al. [72] 2003	F4863_D4869delinsT	Clinical and functional effects of a deletion in a COOH-terminal lumenal loop of the skeletal muscle ryanodine receptor	Channels carrying the deletion were less stable than the wild-type channels and disappeared rapidly when recorded at membrane potentials greater than ±20 mV
Stange M, et al. [73] 2003	S2843D, S2843A	Characterization of recombinant skeletal muscle (Ser-2843) and cardiac muscle (Ser-2809) ryanodine receptor phosphorylation mutants	Results did not support the view that phosphorylation of a single site (RyR1-Ser-2843 and RyR2-Ser-2809) substantially changes RyR1 and RyR2 channel function
Loke JC, et al. [74] 2003	R328W	Detection of a novel ryanodine receptor subtype 1 mutation (R328W) in a malignant hyperthermia family by sequencing of a leukocyte transcript	The mutant channel has increased sensitivity to both caffeine and halothane
Yamaguchi N, et al. [75] 2001	V3619A, W3620A, L3624D, Δ4274–4535	Identification of apocalmodulin and Ca^2+^ − calmodulin regulatory domain in skeletal muscle Ca^2+^ release channel, ryanodine receptor	Two single amino acid substitutions distinctly change the regulation of the skeletal muscle Ca^2+^ release channel by CaM; one of which (L3624D) results in a loss of activation by apoCaM and an inhibition by CaCaM, whereas the other (W3620A) specifically abolishes CaCaM inhibition RyR1Δ4274–4535, showed an ∼10-fold increased sensitivity to activating Ca^2+^
Sun J, et al. [76] 2001	C3635A	Cysteine-3635 is responsible for skeletal muscle ryanodine receptor modulation by NO	C3635A resulted in the loss of CaM-dependent NO modulation of channel activity and reduced S-nitrosylation by NO to background levels but did not affect NO-independent channel modulation by CaM or the redox sensitivity of the channel to O(2) and glutathione
Gaburjakova M, et al. [77] 2001	V2461H, V2461E, V2461G, V2461I	FKBP12 binding modulates ryanodine receptor channel gating	Val2461 is a critical residue required for FKBP12 binding to RyR1 FKBP12 has a functional role in the RyR1 channel complex
Du GG, et al. [78] 2001	G2370A, G2372A, G2373A, G2375A, Y3937A, S3938A, G3939A, K3940A	Mutations to Gly2370, Gly2373 or Gly2375 in malignant hyperthermia domain 2 decrease caffeine and cresol sensitivity of the rabbit skeletal-muscle Ca^2+^ − release channel (ryanodine receptor isoform 1)	Amino acids 2370–2375 lie within a sequence (amino acids 2163–2458) in which 8 RyR1 mutations associated with MH have been shown to be hypersensitive to caffeine and 4-chloro-m-cresol activation By contrast, G2370A, G2373A and G2375A are hyposensitive to caffeine and 4-chloro-m-cresol Amino acids 2163–2458 form a regulatory domain (MH regulatory domain 2) that regulates caffeine and 4-chloro-m-cresol sensitivity of RyR1
Monnier N, et al. [79] 2000	Y4796C	An autosomal dominant congenital myopathy with cores and rods is associated with a neomutation in the *RYR1* gene encoding the skeletal muscle ryanodine receptor	Expression of the mutant RYR1 cDNA produced channels with increased caffeine sensitivity and a significantly reduced maximal level of Ca^2+^ release Single-cell Ca^2+^ analysis showed that the resting cytoplasmic level was increased by 60% in cells expressing the mutant channel
Gao L, et al. [80] 2000	I4897A, I4897L, I4897V, D4917A, D4899A, D4899R, R4913E, G4894A, D4899N	Evidence for a role of the lumenal M3-M4 loop in skeletal muscle Ca^2+^ release channel (ryanodine receptor) activity and conductance	Amino acid residues in the lumenal loop region between the two most C-terminal membrane segments constitute a part of the ion-conducting pore of RyR1
Tong J, et al. [81] 1999	C36R, G249R, G342R, R553W, R615R, R615C, R2163C, G2435R, R2458C, R2458H, R164C, I404M, Y523S, R2163H, R2436H	Measurement of resting cytosolic Ca^2+^ concentrations and Ca^2+^ store size in HEK-293 cells transfected with malignant hyperthermia or central core disease mutant Ca^2+^ release channels	MH/CCD mutants were more sensitive to caffeine than WT RyR1, indicating that caffeine hypersensitivity observed with a variety of MH/CCD mutant RyR1 proteins is not dependent on extracellular Ca^2+^ concentration
Lynch PJ, et al. [82] 1999	I4898T	A mutation in the transmembrane/luminal domain of the ryanodine receptor is associated with abnormal Ca^2+^ release channel function and severe central core disease	Single-cell analysis of co-transfected cells showed a significantly increased resting cytoplasmic Ca^2+^ level and a significantly reduced luminal Ca^2+^ level These data are indicative of a leaky channel, possibly caused by a reduction in the Ca^2+^ concentration required for channel activation
Tong J, et al. [83] 1997	R164C, G249R, G342R, I404M, Y523S, R615C, G2435R, R2436H, C36R, R553W, R615L, R2163C, R2163H, R2458C, R2458H	Caffeine and halothane sensitivity of intracellular Ca^2+^ release is altered by 15 calcium release channel (ryanodine receptor) mutations associated with malignant hyperthermia and/or central core disease	Abnormal sensitivity in the Ca^2+^ photometry assay provides supporting evidence for a causal role in MH for each of 15 single amino acid mutations in the ryanodine receptor

#### Expression of recombinant RYR1 in dyspedic myotubes

Transfection of dyspedic myotubes with mutant *RYR1* cDNA was reported in 25 publications, Table 2, in which a total of 49 unique variations were tested. This includes studies that used the 1B5 cell line, derived by transduction of dyspedic mouse fibroblasts with *MyoD,* to evaluate mutant RyR1 channel function [109]. Of these 49 variations, 44 were missense substitutions and 5 were deletions with a majority (27/49) affecting the RyR1 channel and activation core domain. One missense substitution, E4032A [103, 105, 106], was evaluated and/or functionally characterized at least three times in transfected dyspedic myotubes.

**Table 2:** Cellular *RYR1* model systems: Transfected *RYR1*-null (dyspedic) myotubes

Author/Year	***RYR1*** variant(s)	Title	Conclusions
Dyspedic myotubes			
Lefebvre R et al. [86] 2013	R4892W, G4896V	Ca2+ release in muscle fibers expressing R4892W and G4896V type 1 ryanodine receptor disease mutants	The dominant-negative effect of the R4892W mutant on voltage-gated Ca^2+^ release in myotubes and adult muscle fibers was considerably less than that observed for G4896V
Groom L, et al. [87] 2011	R3983C, D4505H	Identical de novo mutation in the type 1 ryanodine receptor gene associated with fatal, stress-induced malignant hyperthermia in two unrelated families	The functional impact of the two variants expressed in RyR1-nullmyotubes depends on whether the two variants are located on common or separate subunits
Booms P, et al. [88] 2009	E2347del	Concentration dependence of caffeine-induced Ca2+ release in dyspedic skeletal myotubes transfected with ryanodine receptor isoform-1 (RYR1) cDNAs	E2347del increases the sensitivity of RyR1 to caffeine
Yang T, et al. [89] 2007	R164C, R165C, R2163C, T4825I	Elevated resting [Ca(2+)](i) in myotubes expressing malignant hyperthermia RyR1 cDNAs is partially restored by modulation of passive calcium leak from the SR	Myotubes expressing any of the four MH RyR1s (at least 1 from all 3 mutation hot spots) had a higher resting [Ca2+] than those expressing WTRyR1 The elevated resting [Ca2+]i observed in myotubes expressing the four MHRyR1s varied among the individual mutations Treatment of myotubes expressing WT/MHRyR1s with ryanodine or FLA 365 had no effect on resting [Ca2+] Incubation of myotubes with bastadin 5 or ryanodine and bastadin 5 in combination significantly lowered resting [Ca2+]i in myotubes expressing either WTRyR1 and MHRyR1s The percent decrease in resting [Ca2+]i after treatment with bastadin 5 or the combination of ryanodine and bastadin 5 in myotubes expressing MHRyR1s was significantly greater than in myotubes expressing WTRyR1s
Yang T, et al. [90] 2007	R615C, R2163C, T48261	Enhanced excitation-coupled calcium entry in myotubes is associated with expression of RyR1 malignant hyperthermia mutations	RyR1 MH mutations are associated with an enhanced Ca2+ entry through the sarcolemma during depolarization Ca2+ entry may contribute to maintaining Ca2+ homeostasis in mammalian skeletal EC coupling and may play an important role in the pathophysiology of malignant hyperthermia
Goonasekera SA, et al. [91] 2007	D4878A, D4907A, E4908A	Triadin binding to the C-terminal luminal loop of the ryanodine receptor is important for skeletal muscle excitation contraction coupling	triadin binding to RyR1 enhances release channel activity during both voltage and ligand activation and that this critical regulation of release channel activity ensures robust and rapid calcium release during skeletal muscle EC coupling
Lyfenko AD, et al. [66] 2007	R4214_F4216del, V4927_I4928del	Two central core disease (CCD) deletions in the C-terminal region of RYR1 alter muscle excitation-contraction (EC) coupling by distinct mechanisms	R4214_F4216del promotes Ca^2+^ depletion from intracellular stores by exhibiting a classic “leaky channel” behavior V4927_I4928del deletion reduces Ca^2+^ release by disrupting Ca^2+^ gating and eliminating Ca^2+^ permeation through the open channel
Lee EH, et al. [92] 2006	D4878A, D4907A, E4908A	Occurrence of atypical Ca^2+^ transients in triadin-binding deficient-RYR1 mutants	There was similarly atypical Ca^2+^ transients in response to caffeine in myotubes expressing all 3 mutations and the single mutant (D4907A) Differences in triadin-binding and SR Ca^2+^ release observed in this study can be attributed to an alteration in a single amino acid (D4907)
Aracena-Parks P, et al. [93] 2006	C3635A	Identification of cysteines involved in S-nitrosylation, S-glutathionylation, and oxidation to disulfides in ryanodine receptor type 1	12 of the 100 cysteines on RyR1 can be redox-modified and that 9 of these cysteines appear to be endogenously modified to some extent We also show that the different redox agents target some of the same cysteines, but Cys-1040 and Cys-1303 are exclusively S-nitrosylated, whereas Cys-1591 and Cys-3193 are exclusively S-glutathionylated On the other hand, Cys-3635 can be S-nitrosylated, S-glutathionylated, or oxidized to form a disulfide and also influences Ca^2+^ release during EC coupling
Hurne AM, et al. [94] 2005	C4958S, C4961S	Ryanodine receptor type 1 (RyR1) mutations C4958S and C4961S reveal excitation-coupled calcium entry (ECCE) is independent of sarcoplasmic reticulum store depletion	There is an essential role of Cys(4958) and Cys(4961) within an invariant CXXC motif for stabilizing conformations of RyR1 that influence both its function as a release channel and its interaction with ECCE channels
Cheng W, et al. [95] 2005	D3490_N3523del	Interaction between the dihydropyridine receptor Ca2+ channel β-subunit and ryanodine receptor type 1 strengthens excitation-contraction coupling	EC coupling in skeletal muscle involves the interplay of at least two subunits of the DHPR, namely alpha1S and beta1a, interacting with possibly different domains of RyR1
Du GG, et al. [96] 2004	4274_4535del	Role of the sequence surrounding predicted transmembrane helix M4 in membrane association and function of the Ca(2+) release channel of skeletal muscle sarcoplasmic reticulum (ryanodine receptor isoform 1)	Maximal amplitudes of L-currents and Ca^2+^ transients with Delta4274–4535 were larger than with wild-type RyR1, and voltage-gated sarcoplasmic reticulum Ca^2+^ release was more sensitive to activation by sarcolemmal voltage sensors Thus, this region may act as a negative regulatory module that increases the energy barrier for Ca^2+^ release channel opening
Dirksen RT, et al. [97] 2004	Y4795C, R2435L, R2163H	Distinct effects on Ca2+ handling caused by malignant hyperthermia and central core disease mutations in RyR1	MH-only mutations modestly increase basal release-channel activity in a manner insufficient to alter net SR Ca^2+^ content (“compensated leak”), whereas the mixed MH + CCD phenotype arises from mutations that enhance basal activity to a level sufficient to promote SR Ca^2+^ depletion, elevate [Ca^2+^], and reduce maximal VGCR (“decompensated leak”)
Zhu X, et al. [98] 2004	3614_3643del	The calmodulin binding region of the skeletal ryanodine receptor acts as a self-modulatory domain	Depolarization-, caffeine- and 4-chloro-m-cresol (4-CmC)-induced Ca^2+^ transients in these cells were dramatically reduced compared with cells expressing WT RyR1. Deletion of the 3614–3643 region resulted in profound changes in unitary conductance and channel gating RyR1 3614–3643 region acts not only as the CaM binding site, but also as an important modulatory domain for RyR1 function
Yang T, et al. [99] 2003	R163C, G341R, R614C, R2163C, V2168M, R2458H, T4826I	Functional defects in six ryanodine receptor isoform-1 (RyR1) mutations associated with malignant hyperthermia and their impact on skeletal excitation-contraction coupling	These 7 MH mutations are all both necessary and sufficient to induce MH-related phenotypes Decreased sensitivity to Ca^2+^ and Mg^2+^ inhibition and inability of MHRyR1s to be fully inactivated at [Ca^2+^] typical of normal myotubes at rest are key defects that contribute to the initiation of MH episodes
Avila G, et al. [100] 2003	Y523S, Y4795C, I4897T, G4890R, R4892W, G4898E, G4898R, A4905V, R4913G	The pore region of the skeletal muscle ryanodine receptor is a primary locus for excitation-contraction uncoupling in central core disease	CCD mutations in exon 102 disrupt release channel permeation to Ca^2+^ during EC coupling and that this region represents a primary molecular locus for EC uncoupling in CCD
Avila G, et al. [101] 2003	V2461G, V2461I	FKBP12 binding to RyR1 modulates excitation-contraction coupling in mouse skeletal myotubes	None of the mutations that disrupted FKBP binding to RyR1 significantly affected RyR1-mediated enhancement of L-type Ca^2+^ channel activity (retrograde coupling) FKBP12 binding to RyR1 enhances the gain of skeletal muscle EC coupling
O’Connell KM, et al. [102] 2002	L3624D, W3620A	Calmodulin binding to the 3614–3643 region of RyR1 is not essential for excitation-contraction coupling in skeletal myotubes	Expression of either L3624D or W3620A in dyspedic myotubes restored both L-type Ca^2+^ currents (retrograde coupling) and voltage-gated SR Ca^2+^ release (orthograde coupling) to a similar degree as that observed for wild-type RyR1, although L-current density was somewhat larger and activated at more hyperpolarized potentials in W3620A-expressing myotubes CaM binding to the 3614–3643 region of RyR1 is not essential for voltage sensor activation of RyR1
O’Brien JJ, et al. [103] 2002	E4032A	Ca2+ activation of RyR1 is not necessary for the initiation of skeletal-type excitation-contraction coupling	Depolarization of E4032A-RyR1-expressing myotubes elicited L-type Ca^2+^ currents of approximately normal size and myoplasmic Ca^2+^ transients that were skeletal-type, but about fivefold smaller than those for wild-type RyR1 The reduced amplitude of the Ca^2+^ transient is consistent either with the possibility that Ca^2+^ activation amplifies Ca^2+^ release during EC coupling, or that the E4032A mutation generally inhibits activation of RyR1 Ca^2+^ activation of RyR1 does not appear to be necessary for the initiation of Ca^2+^ release during EC coupling in skeletal muscle
Feng W, et al. [104] 2002	F1777R, F1782R	Homer regulates gain of ryanodine receptor type 1 channel complex	1B5 dyspedic myotubes expressing RyR1 with a point mutation of a putative Homer-binding domain exhibit significantly reduced (approximately 33%) amplitude in their responses to K^+^ depolarization compared with cells expressing wild type protein These results reveal that in addition to its known role as an adapter protein, Homer is a direct modulator of Ca^2+^ release gain
Fessenden JD, et al. [105] 2001	E4032A	Ryanodine receptor point mutant E4032A reveals an allosteric interaction with ryanodine	Results with the E4032A mutant channel suggest that ryanodine does not act as a pore blocker but instead, that ryanodine binding sites reside outside of the permeation pore, and that ryanodine binding to these sites has allosteric effects on calcium permeability
Avila G, et al. [106] 2001	E4032A	Ca^2+^ release through ryanodine receptors regulates skeletal muscle L-type Ca^2+^ channel expression	Long-term expression of E4032A, a mutant RyR-1 that preferentially affects the orthograde signal of E-C coupling (i.e., fully restores L-channel activity but not SR Ca^2+^ release) failed to increase functional DHPR expression
Avila G, et al. [107] 2001	I4897T	Excitation - Contraction uncoupling by a human central core disease mutation in the ryanodine receptor	Muscle weakness suffered by individuals possessing the I4898T mutation involves a functional uncoupling of sarcolemmal excitation from SR Ca^2+^ release, rather than the expression of overactive or leaky SR Ca^2+^ release channel
Avila G, et al. [108] 2001	R164C, I404M, Y523S, R2163H, R2435H	Functional effects of central core disease mutations in the cytoplasmic region of the skeletal muscle ryanodine receptor	Resting Ca^2+^ levels were elevated in dyspedic myotubes expressing four of these mutants (Y523S > R2163H > R2435H R164C > I404M RyR1)

#### Expression of endogenous mutant RYR1

Additionally, 16 publications reported immortalization of patient primary B-lymphocytes for downstream functional characterization, Table 3. These 16 publications included 32 unique missense substitutions, one deletion, and two deletion-insertions. A total of 50 unique *RYR1* variants, all missense substitutions, were tested in 19 publications utilizing primary cell culture model systems, Table 4.

**Table 3:** Cellular *RYR1* model systems: Immortalized B-lymphocytes

Author/Year	***RYR1*** variant(s)	Title	Conclusions
Zullo A, et al. [110] 2019	R1335C, S2345R, S3098I, F4924_V4925ins	*RYR1* sequence variants in myopathies: Expression and functional studies in two families	Ca^2+^ release in response to the RyR1 agonist 4-chloro-m-cresol and to thapsigargin showed that S2345R causes depletion of S/ER Ca^2+^ stores and that the compound heterozygosity for variant S3098I and the 30-nucleotide insertion increases RyR1-dependent Ca^2+^ release without affecting ER Ca^2+^ stores
Johannsen S, et al. [111] 2016	R4737W	Functional characterization of the RYR1 mutation pArg4737Trp associated with susceptibility to malignant hyperthermia	Intracellular resting calcium was slightly but significantly elevated in mutation positive cells. Calcium release following stimulation with 4-chloro-m-cresol was significantly increased in B lymphocytes carrying the R4737W mutation compared to mutation negative controls
Schiemann AH, et al. [112] 2014	R2355W, V2354M	Functional characterization of 2 known ryanodine receptor mutations causing malignant hyperthermia	We propose that R2355W is confirmed as being an MH-causative mutation and suggest that V2354M is a *RYR1* mutation likely to cause MH
Lyfenko AD, et al. [66] 2007	R4214_F4216del	Two central core disease (CCD) deletions in the C-terminal region of RYR1 alter muscle excitation-contraction (EC) coupling by distinct mechanisms	ΔRQF *RYR1* deletion did not significantly affect the sensitivity of lymphoblastoid cells to activation by 4-CmC. However, Ca21 release activated by a maximal concentration of 4-CmC (1 mM) was significantly reduced in ΔRQF-carrying cells
Schiemann AH, et al. 2013	R1583C, V2102L	Sequence capture and massively parallel sequencing to detect mutations associated with malignant hyperthermia	The amount of Ca^2+^ released after stimulation with 4-chloro-m-cresol from B lymphocytes of the MH-susceptible patients in the family was significantly greater compared with that of Ca^2+^ released from cells of an MH-negative family member
Attali R, et al. [113] 2013	Y3016C	Variable myopathic presentation in a single family with novel skeletal RYR1 mutation	Functional analysis on EBV immortalized cell lines showed no effect of the mutation on RyR1 pharmacological activation or the content of intracellular Ca^2+^ stores
Vukcevic M, et al. [114] 2010	R1679H, K1393R, E1058K, H382N, R2508G	Functional properties of RYR1 mutations identified in Swedish patients with malignant hyperthermia and central core disease	All B lymphoblastoid cell lines carrying RYR1 candidate mutations showed significantly increased resting cytoplasmic Ca^2+^ levels as well as a shift to lower concentrations of 4-CmC inducing calcium release compared with controls
Grievink H, et al. [115] 2010	H4833Y	Allele-specific differences in ryanodine receptor 1 mRNA expression levels may contribute to phenotypic variability in malignant hyperthermia	Allele-specific differences in RYR1 mRNA expression levels in heterozygous MHS samples, and can at least in part contribute to the observed variable penetrance and variations in MH clinical phenotypes
Zozato F, et al. [72] 2003	F4863_D4869delinsT	Clinical and functional effects of a deletion in a COOH-terminal lumenal loop of the skeletal muscle ryanodine receptor	Cells have depleted thapsigargin-sensitive intracellular Ca^2+^ stores, exhibit release of Ca^2+^ from intracellular stores in the absence of the addition of a pharmacological activator of the RYR1; and the unelicited Ca^2+^ transient from the thapsigargin-sensitive stores could be blocked by dantrolene, a specific inhibitor of the skeletal muscle RYR
Zullo A, et al. [116] 2009	R530H, R2163P, N2342S, E2371G, R2454H, C4664R	Functional characterization of ryanodine receptor (RYR1) sequence variants using a metabolic assay in immortalized B-lymphocytes	Increased acidification rate of lymphoblastoid cells in response to 4-CmC is mainly due to RYR1 activation. Cells expressing RYR1 variants in the N-terminal and in the central region of the protein (R530H, R2163P, N2342S, E2371G and R2454H) displayed higher activity compared with controls. Cell lines harboring RYR1(C4664R) were significantly less activated by 4-CmC
Levano S, et al. [117] 2009	D554Y, R2336H, E2404K, D2730G	Increasing the number of diagnostic mutations in malignant hyperthermia	All *RYR1* mutations significantly increased resting calcium concentration and significantly affect either 4-CmC or caffeine dose-response curve to pharmacological activation Only one mutation (D2730G) showed a significant reduction in EC50 of both caffeine and 4-CmC
Anderson AA, et al. [118] 2008	H4833Y	Identification and biochemical characterization of a novel ryanodine receptor gene mutation associated with malignant hyperthermia	B lymphocytes from patients with this mutation were approximately twofold more sensitive than MH-negative cells to activation with 4-CmC. The amount of Ca^2+^ released from B lymphocytes of MH-susceptible patients was significantly greater than that released from cells of family members without this mutation
Ducreux D, et al. [119] 2006	P3527S, V4849I, R999H	Functional properties of ryanodine receptors carrying three amino acid substitutions identified in patients affected by multi-minicore disease and central core disease, expressed in immortalized lymphocytes	P3527S in the homozygous state affected the amount of Ca^2+^ released after pharmacological activation with 4-CmC and caffeine but did not affect the size of the thapsigargin-sensitive Ca^2+^ stores. The other substitutions had no effect on the size of the intracellular Ca^2+^ stores, or the amount of Ca^2+^ released after ryanodine receptor activation P3527S and V4849I substitutions had a small but significant effect on the resting Ca^2+^ concentration
Tilgen N, et al. [120] 2001	R4861H, I4898T, G4899A	Identification of four novel mutations in the C-terminal membrane spanning domain of the ryanodine receptor 1: Association with central core disease and alteration of calcium homeostasis	Cell showed release of Ca^2+^ from intracellular stores in the absence of any pharmacological activators of RYR, significantly smaller thapsigargin-sensitive intracellular calcium stores, compared to lymphoblasts from control individuals, and a normal sensitivity of the calcium release to the RYR inhibitor dantrolene
Girard T, et al. [121] 2001	V2168M	B-lymphocytes from malignant hyperthermia-susceptible patients have an increased sensitivity to skeletal muscle ryanodine receptor activators	EBV immortalized cells harboring the V2168M RYR1 gene mutation were more sensitive to the RYR activator 4-CmC and their peripheral blood leukocytes produce more interleukin-1beta after treatment with the RYR activators caffeine and 4-CmC, compared with cells from healthy controls
Hoppe K, et al. [122] 2016	R530H, C4664R, R2163P	Hypermetabolism in B–lymphocytes from malignant hyperthermia susceptible individuals	Native B–lymphocytes from MHS individuals are more sensitive to 4–CmC than those from MHN, reflecting a greater Ca^2+^ turnover. The acidification response, however, was less pronounced than in muscle cells, presumably reflecting the lower expression of RyR1 in B–lymphocytes.

**Table 4:** Primary cell culture model systems

Author/Year	Species/***RYR1*** variant(s)	Title	Conclusions
Suman M, et al. [123] 2018	N4575T, I1571V, L3136Rfs, R163C, I4898T, Q4837RfsX3	Inositol trisphosphate receptor-mediated Ca^2+^ signaling stimulates mitochondrial function and gene expression in core myopathy patients	Remodeling of skeletal muscle Ca^2+^ signaling following loss of functional RyR1 mediates bioenergetic adaptation
Choi RH, et al. [124] 2017	R1976C	Dantrolene requires Mg^2+^ to arrest malignant hyperthermia	Accumulation of the metabolite Mg^2+^ from MgATP hydrolysis is required to make dantrolene administration effective in arresting an MH episode
Hoppe K, et al. [122] 2016	G2434R, R614C	Hypermetabolism in B-lymphocytes from malignant hyperthermia susceptible individuals	Native B–lymphocytes from MHS individuals are more sensitive to 4–CmC than those from MHN, reflecting a greater Ca^2+^ turnover. The acidification response, however, was less pronounced than in muscle cells, presumably reflecting the lower expression of RyR1 in B–lymphocytes
Kaufmann A, et al. [125] 2012	A612P, R2458H, R3348C	Novel double and single ryanodine receptor 1 variants in two Austrian malignant hyperthermia families	Results suggest that these variants are new causative MH variants
Treves S, et al. [126] 2010	V2168M, R2336H, R614C, D2730G, R44C, R789L	Enhanced excitation-coupled Ca^2+^ entry induces nuclear translocation of NFAT and contributes to IL-6 release from myotubes from patients with central core disease	Excitation-coupled calcium entry is strongly enhanced in cells from patients with CCD compared with individuals with MH and controls. Excitation-coupled calcium entry induces generation of reactive nitrogen species and enhances nuclear localization of NFATc1, which in turn may be responsible for the increased IL-6 released by myotubes from patients with CCD
Kobayashi M, et al. [127] 2011	L4838V, R2508C	Analysis of human cultured myotubes responses mediated by ryanodine receptor 1	Among samples from CICR-accelerated patients, there was an increased sensitivity to RYR1 activators compared to non-accelerated patients. The EC50 values for these different compounds correlated with results of CICR testing. Using this approach may be a sensitive and specific method of identifying patients predispose to MH
Migita T, et al. [62] 2009	R2508C, A4894T	Do Ca^2+^ channel blockers improve malignant hyperthermia crisis?	The dantrolene-induced decline effect of Ca^2+^ of skeletal muscle was not disappeared in the presence of Ca^2+^ blockers. In MH crisis, we do not recommend to administer Ca^2+^ blockers because of its potent effect to increase Ca^2+^
Migita T, et al. [128, 129] 2007	R2508C, L4838V	Propofol-Induced Changes in Myoplasmic Calcium Concentrations in Cultured Human Skeletal Muscles from RYR1 Mutation Carriers	Increases in calcium concentrations in response to propofol dosage were limited to doses at least 100-fold greater than those used in clinical settings. These observations correlate well with clinical observations that propofol does not trigger malignant hyperthermia in susceptible humans
Zhou, et al. [130] 2006	R109W, M402T, M2423K, R2939K, A4329D, T4709M	Epigenetic allele silencing unveils recessive RYR1 mutations in core myopathies	RYR1 undergoes polymorphic, tissue-specific, and developmentally regulated allele silencing and that this unveils recessive mutations in patients with core myopathies
Weigl LG, et al. [131] 2004	G2434R	4-Chloro-m-cresol cannot detect malignant hyperthermia equivocal cells in an alternative minimally invasive diagnostic test of malignant hyperthermia susceptibility	Cells of MHEH individuals showed low sensitivities against both caffeine and 4-CmC, comparable to those of the MHN group. Therefore, with myotubes, caffeine was able to discriminate between MHS and MHN cells, but both caffeine and 4-CmC failed to detect MHEH cells
Wehner M, et al. [132] 2004	A2350T, R2355W, G2375A	Functional characterization of malignant hyperthermia-associated RyR1 mutations in exon 44, using the human myotube model	Investigation of calcium homeostasis with the calcium sensitive probe Fura 2 showed a higher sensitivity to the ryanodine receptor agonists 4-chloro-m-cresol, caffeine and halothane for the myotubes derived from the mutation carriers as compared to those of the control group
Ducreux S, et al. [133] 2004	V2168M, I4898T, R4893W	Effect of ryanodine receptor mutations on interleukin-6 release and intracellular calcium homeostasis in human myotubes from malignant hyperthermia-susceptible individuals and patients affected by central core disease	Abnormal release of calcium via mutated RYR enhances the production of the inflammatory cytokine IL-6, which may in turn affect signaling pathways responsible for the trophic status of muscle fibers
Wehner M, et al. [134] 2003	I2182F, G2375A	Calcium release from sarcoplasmic reticulum is facilitated in human myotubes derived from carriers of the ryanodine receptor type 1 mutations Ile2182Phe and Gly2375Ala	In myotubes of individuals carrying the RyR1 Ile2182Phe or the RyR1 Gly2375Ala mutation, the EC(50) for caffeine and halothane was reduced; in the Ile2182Phe myotubes, the EC(50) for 4CmC was also reduced, all consistent with facilitated calcium release from the sarcoplasmic reticulum. From these data we conclude that both mutations are pathogenic for MH
Wehner M, et al. [135] 2003	I2453T	The Ile2453Thr mutation in the ryanodine receptor gene 1 is associated with facilitated calcium release from sarcoplasmic reticulum by 4-chloro-m-cresol in human myotubes	The reduction of EC(50) indicates a facilitated calcium release from sarcoplasmic reticulum in the myotubes of the index patient suggesting that the RYR1 Ile2453Thr mutation is pathogenic for the malignant hyperthermia susceptibility and CCD of the two affected individuals
Wehner M, et al. [136] 2002	T2206M	Increased sensitivity to 4-chloro-m-cresol and caffeine in primary myotubes from malignant hyperthermia susceptible individuals carrying the ryanodine receptor 1 Thr2206Met (C6617T) mutation	In myotubes the half-maximal activation concentration (EC(50)) for 4-chloro-m-cresol was reduced from 203 micro m (wild type) to 98 micro m (Thr2206Met), and for caffeine from 3.8 mm to 1.8 mm. From the reduction of EC(50) we conclude that the RyR1 Thr2206Met mutation is pathogenic for MH
Sei Y, et al. [137] 2002	C35R, R163C, G248R, G341R, I403M, R552W, R614C, R614L, R2163C, R2163H, V2168, V2214I, A2367T, D2431N, G2434R, R2435H, R2454C, R2454H, R2458C, R2458H, I4898T	Patients with malignant hyperthermia demonstrate an altered calcium control mechanism in B lymphocytes	The Ca^2+^ responses to caffeine or 4-chloro-m-cresol in B lymphocytes showed significant differences between MHS and MHN (or control) individuals. Although the molecular mechanisms of these alterations are currently undetermined, the results suggest that the enhanced Ca^2+^ responses are associated with mutations in the RYR1 gene in some MHS individuals
Girard T, et al. [138] 2002	R614C, G2434R, V2168M, R2458C	Phenotyping malignant hyperthermia susceptibility by measuring halothane-induced changes in myoplasmic calcium concentration in cultured human skeletal muscle cells	Measurements of Ca^2+^ in human skeletal muscle cells can be used to phenotype MH susceptibility; however, we did not observe a specific effect of any mutation in the RYR1 gene on the halothane-induced increase in Ca^2+^
Brinkmeier H, et al. [139] 1999	G2435R	Malignant hyperthermia causing Gly2435Arg mutation of the ryanodine receptor facilitates ryanodine-induced calcium release in myotubes	The phenotype of MH can be characterized using cultured human muscle and a culture-based test for MH susceptibility may eventually be developed.
Censier K, et al. [140] 1998	R163C	Intracellular calcium homeostasis in human primary muscle cells from malignant hyperthermia-susceptible and normal individuals. Effect Of overexpression of recombinant wild-type and Arg163Cys mutated ryanodine receptors	Cultured human skeletal muscle cells derived from MH-susceptible individuals exhibit a half-maximal halothane concentration causing an increase in intracellular Ca^2+^ concentration which is twofold lower than that of cells derived from MH-negative individuals. The resting Ca^2+^ concentration of cultured skeletal muscle cells from MH-negative and MH-susceptible individuals is not significantly different

### Animal model systems

#### Mice

A total of 15 *RYR1* rodent model systems were identified of which ten were heterozygous, three were compound heterozygous, and a further two were knockout, Table 5. Variations discussed in this section are numbered according to the mouse sequence. Core formation was reported in three of the rodent model systems, excluding knockout (Y524S [158], Q1970fsX16 + A4329D [179], I4895T [168]). Overall, six of the ten heterozygous rodent model systems had missense substitutions affecting the RyR1 cytosolic shell domain. Two compound heterozygous model systems had a single missense substitution engineered into one allele with a frameshift leading to a deletion or truncation on the opposite allele [178, 179]. In these model systems, one variation affected the RyR1 cytosolic shell and the other affected the RyR1 channel and activation core. An additional compound heterozygous model system had a single missense substitution affecting the RyR1 channel and activation core with a second missense substitution and deletion, on the opposite allele, affecting the RyR1 cytosolic shell [181]. Various forms of aberrant intracellular calcium dynamics were reported in all rodent systems (except knockout). This included evidence of increased resting cytosolic calcium and RyR1-open probability under resting conditions (SR calcium leak) [173] as well as decreased calcium permeation (excitation-contraction uncoupling) [30]. The two most frequently reported *RYR1* rodent model systems were the dyspedic mouse, accounting for 47% of rodent publications [54, 109, 194–238], and the Y524S knock-in mouse, which accounted for 22% of rodent publications, Table 5. Studies utilizing dyspedic mice/1B5 myotubes not transfected with mutant *RYR1* cDNA, were primarily focused on elucidating the following: (a) relative importance and functional role of wild-type RyR isoforms [213, 227, 234], (b) fundamental physiology of excitation-contraction coupling components [205, 225, 232], (c) roles of specific RyR1 structural regions on channel function [216, 222, 235]. The Y524S knock-in mouse has been utilized extensively to investigate the mechanisms behind several phenotypes on the *RYR1*-RM disease spectrum including MH susceptibility [162], statin-induced myopathy [152], and central core disease [158]. Y524S mice have also been used to test potential therapeutics for *RYR1*-RM including the antioxidant *N*-acetylcysteine [145, 160] and the activator of the AMP-activated protein kinase 5-aminoimidazole-4-carboxamide ribonucleoside (AICAR) [153].

**Table 5:** Rodent *RYR1* model systems

Author/Year	***Ryr1*** variant(s)	Title	Conclusions
YS mouse (equivalent to Y522S in humans)			
Zullo A, et al. [141] 2018	Y524S	Voltage modulates halothane-triggered Ca^2+^ release in malignant hyperthermia-susceptible muscle	Binding of halothane to RyR1 alters the voltage dependence of Ca^2+^ release in MH-susceptible muscle fibers such that the resting membrane potential becomes a decisive factor for the efficiency of the drug to trigger Ca^2+^ release
O-Uchi J, et al. [142] 2017	Y524S	Malignant hyperthermia-associated mutation of leaky RyR1 induces mitochondrial Ca^2+^ overload in the heart	Chronic mitochondrial Ca^2+^ overload via leaky mutant mRyR1 damages cardiac mitochondrial functions/structures, which may alter cytosolic Ca^2+^ handling, induce cellular oxidation, and increase the arrhythmogenic events in MH
Abeele FV, et al. [143] 2019	Y524S	TRPV1 variants impair intracellular Ca^2+^ signaling and may confer susceptibility to malignant hyperthermia	Trpv1 may be contributing to the mechanism underlying the hyperthermia response of this Y524S Ryr1 model TRPV1 and related mutants could be a new therapeutic target for treating muscle diseases due to altered regulation of Ca^2+^ release
Michelucci A, et al. [144] 2017	Y524S	Strenuous exercise triggers a life-threatening response in mice susceptible to malignant hyperthermia	Strenuous physical exertion triggers lethal episodes in MH-susceptible mice and these episodes share common features with MH episodes triggered by anesthetics and heat (ie, hyperthermia and rhabdomyolysis)
Michelucci A, et al. [145] 2017	Y524S	Antioxidant Treatment Reduces Formation of Structural Cores and Improves Muscle Function in RYR1(Y522S/WT) Mice	NAC administration is beneficial to prevent mitochondrial damage and formation of cores and improve muscle function in RYR1Y522S/WT mice
Lopez RJ, et al. [146] 2016	Y524S	An RYR1 mutation associated with malignant hyperthermia is also associated with bleeding abnormalities	Y522S mice had longer bleeding times than their WT littermates. Primary vascular smooth muscle cells from Y524S mice exhibited a higher frequency of subplasmalemmal Ca^2+^ sparks, leading to a more negative resting membrane potential. The bleeding defect of Y524S mice and of one patient was reversed by treatment with the RYR1 antagonist dantrolene, and Ca^2+^ sparks in primary vascular smooth muscle cells from Y524S mice were blocked by ryanodine or dantrolene
O-Uchi J, et al. [147] 2016	Y524S	Malignant hyperthermia-associated mutation of RyR1 induces mitochondrial damages and cellular oxidation in the heart	Chronic mitochondrial Ca^2+^ overload via leaky mutant mRyR1 damages cardiac mitochondrial functions/structures, reduces cytosolic Ca^2+^ buffering capacity and induces cellular oxidation, which may increase arrhythmogenic events in MH
O-Uchi J, et al. [148] 2014	Y524S	RyR1 mutation associated with malignant hyperthermia facilitates catecholaminergic stress-included arrhythmia via mitochondrial injury and oxidative stress	Chronic mitochondrial damage by Ca^2+^ overload via leaky mutant RyR1 induces cellular oxidation, which facilitates catecholaminergic stress-triggered arrhythmia
Yarotskyy V, et al. [149] 2013	Y524S	Accelerated activation of SOCE current in myotubes from two mouse models of anesthetic- and heat-induced sudden death	While an increased rate of SOCE current activation is a common characteristic of myotubes derived from Y524S/+ and dCasq-null mice and that the protective effects of azumolene are not due to a direct inhibition of SOCE channels
Vukcevic M, et al. [150] 2013	Y524S	Gain of function in the immune system caused by a ryanodine receptor 1 mutation	Y524S mice have a gain in immune functions. Gain-of-function MH-linked RYR1 mutations might offer selective immune advantages to their carriers
Manno C, et al. [151] 2013	Y524S	Altered Ca^2+^ concentration, permeability and buffering in the myofibre Ca^2+^ store of a mouse model of malignant hyperthermia	Y524S mutation causes greater openness of the RyR1, lowers resting SR Ca^2+^ and alters SR Ca^2+^ buffering in a way that copies the functional instability observed upon reduction of calsequestrin content
Knoblauch M, et al. [152] 2013	Y524S	Mice with RyR1 mutation (Y524S) undergo hypermetabolic response to simvastatin	An acute dose of simvastatin triggers a hypermetabolic response in YS mice. In isolated YS muscle fibers, simvastatin triggers an increase in cytosolic Ca^2+^ levels by increasing Ca^2+^ leak from the sarcoplasmic reticulum (SR). With higher simvastatin doses, a similar cytosolic Ca^2+^ increase occurs in wild type (WT) muscle fibers. Pre-treatment of YS and WT mice with AICAR prevents the response to simvastatin
Lanner JT, et al. [153] 2012	Y524S	AICAR prevents heat-induced sudden death in RyR1 mutant mice independent of AMPK activation	AICAR is probably effective in prophylactic treatment of humans with enhanced susceptibility to exercise- and/or heat-induced sudden death associated with RYR1 mutations
O-Uchi J, et al. [154] 2012	Y524S	Malignant hyperthermia mutation of RYR1 (Y522S) increases catecholamine-induced cardiac arrhythmia through mitochondrial injury	Chronic mitochondrial damage by Ca^2+^ overload through leaky mutant RyR1 induces mitochondrial structural and functional disruption, which facilitates arrhythmogenic outbursts under acute catecholaminergic stress
Loy RE, et al. [155] 2012	Y524S	Allele-specific gene silencing in two mouse models of autosomal dominant skeletal myopathy	The temperature-dependent increase in resting Ca^2+^ observed in FDB fibers from YS/+ mice was normalized to WT levels after 2 weeks of treatment with YS allele-specific siRNA
Wei L, et al. [156] 2011	Y524S	Mitochondrial superoxide flashes: metabolic biomarkers of skeletal muscle activity and disease	Uncontrolled mitochondrial superoxide production likely contributes to the pathogenic temperature-dependent increase in oxidative stress of RYR1Y524S/WT MH mice
Corona BT, et al. [157] 2010	Y524S	Effect of prior exercise on thermal sensitivity of malignant hyperthermia-susceptible muscle	Eccentric, but not concentric, exercise attenuated the thermal sensitivity of MH-susceptible muscle
Boncompagni S, et al. [158] 2009	Y524S	Characterization and temporal development of cores in a mouse model of malignant hyperthermia	Initial mitochondrial/SR disruption in confined areas causes significant loss of local Ca^2+^ sequestration that eventually results in the formation of contractures and progressive degradation of the contractile elements
Andronache Z, et al. [159] 2009	Y524S	A retrograde signal from RyR1 alters DHP receptor inactivation and limits window Ca^2+^ release in muscle fibers of Y522S RyR1 knock-in mice	The increase in uncompensated SR Ca^2+^ leak observed at rest following transient overexpression of the Y524S RyR1 mutant in myotubes is effectively suppressed after long-term expression of a normal compliment of wild-type and mutant RyR1s in adult muscle fibers of WT/Y524S mice
Durham WJ, et al. [160] 2008	Y524S	RyR1 S-nitrosylation underlies environmental heat stroke and sudden death in Y522S RyR1 knockin mice	Ca^2+^ release channels in RyR1Y524S/wt mice are leaky, producing elevations in resting Ca^2+^, ROS, RNS and basal stress at physiologically relevant temperatures. Ca^2+^ leak enhances RNS production, and subsequent S-nitrosylation of RyR1 further increases Ca^2+^ leak, resulting in regenerative Ca^2+^ release that underlies uncontrolled contractions during heat stress
Corona BT, et al. [161] 2008	Y524S	Eccentric contractions do not induce rhabdomyolysis in malignant hyperthermia susceptible mice	RYR1Y524S/wt protects skeletal muscle from exercise-induced muscle injury. Findings do not support a direct association between MH susceptibility and contraction-induced rhabdomyolysis when core temperature is maintained at lower physiological temperatures during exercise
Chelu MG, et al. [162] 2006	Y524S	Heat- and anesthesia-induced malignant hyperthermia in an RyR1 knock-in mouse	Heterozygous expression of the Y524S mutation confers susceptibility to both heat- and anesthetic-induced MH responses
IT mouse (equivalent to I4898T in humans)			
Lee CS, et al. [163] 2017	I4895T	A chemical chaperone improves muscle function in mice with a RyR1 mutation	Persistent ER stress/UPR, decreased protein synthesis, mitochondrial ROS production/damage and elevation of proapoptotic markers are defining features of RyR1 myopathy associated with the I4895T mutation in mice, making this myopathy distinct from that of the RyR1 myopathies that arise from Ca^2+^ leak. Chemical chaperones and ER stress inhibitors may be better suited for mutations in RyR1 that produce ER stress/UPR
Zvaritch E, et al. [164] 2015	I4895T	Muscle spindles exhibit core lesions and extensive degeneration of intrafusal fibers in the Ryr1(I4895T/wt) mouse model of core myopathy	Muscle spindles undergo severe deterioration that may precede structural changes in extrafusal myofibers Muscle spindles represent an important early target in Ryr1-related disease pathology
De Crescenzo V, et al. [165] 2012	I4895T	Type 1 ryanodine receptor knock-in mutation causing central core disease of skeletal muscle also displays a neuronal phenotype	RyR1 plays a role in voltage-induced Ca^2+^ release in hypothalamic nerve terminals and a neuronal alteration accompanies the myopathy in IT/+ mice
Loy RE, et al. [155] 2012	I4895T	Allele-specific gene silencing in two mouse models of autosomal dominant skeletal myopathy	Altered RyR1 function in FDB fibers of YS/+ and IT/+ knock-in mice can be normalized only two weeks after local in vivo delivery of ASGS siRNAs
Loy RE, et al. [166] 2011	I4895T	Muscle weakness in Ryr1I4895T/WT knock-in mice as a result of reduced ryanodine receptor Ca^2+^ ion permeation and release from the sarcoplasmic reticulum	In vivo muscle weakness observed in IT/+ knock-in mice arises from a reduction in the magnitude and rate of RYR1 Ca^2+^ release during EC coupling that results from the mutation producing a dominant-negative suppression of RYR1 channel Ca^2+^ ion permeation
Boncompagni S, et al. [167] 2010	I4895T	The I4895T mutation in the type 1 ryanodine receptor induces fiber-type specific alterations in skeletal muscle that mimic premature aging	Muscle fibers from IT/+ mice in a mixed 129S6/SvEvTac and 129S2/SvPasCrl background exhibit structural alterations of the type seen in CCD patients as well as in WT mice at older ages
Zvaritch E, et al. [168] 2009	I4895T	Ca^2+^ dysregulation in Ryr1(I4895T/wt) mice causes congenital myopathy with progressive formation of minicores, cores, and nemaline rods	The IT/+ mouse line represents a unique and phenotypically valid model of RyR1-related congenital myopathy with minicores, cores, and rods
Zvaritch E, et al. [30] 2007	I4895T	An Ryr1I4895T mutation abolishes Ca^2+^ release channel function and delays development in homozygous offspring of a mutant mouse line	IT/IT mice, in which RyR1-mediated Ca^2+^ release is abolished without altering the formation of the junctional DHPR-RyR1 macromolecular complex, provide a valuable model for elucidation of the role of RyR1-mediated Ca^2+^ signaling in mammalian embryogenesis
RC mouse (equivalent in humans)			
Truong KM, et al. [169] 2019	R163C	Comparison of Chlorantraniliprole and Flubendiamide Activity Toward Wild-Type and Malignant Hyperthermia-Susceptible Ryanodine Receptors and Heat Stress Intolerance	Although nM-μM of either diamide is capable of differentially altering WT and MHS RyR1 conformation in vitro, human RyR1 mutations within putative diamide N- and C-terminal interaction domains do not alter heat stress intolerance in vivo
Eltit JM, et al. [170] 2013	R163C	Nonspecific sarcolemmal cation channels are critical for the pathogenesis of malignant hyperthermia	nonselective sarcolemmal cation permeability, separate from the classic STIM/Orai pathway, is activated by SR depletion and plays a critical role in the causing cytosolic Ca^2+^ and Na + overload both at rest and during the MH crisis
Estève E, et al. [171] 2012	R163C	Malignant hyperthermia mutation alters excitation-coupled Ca^2+^ entry in MH RyR1-R163C knock-in myotubes	Conformational changes induced by the R163C MH mutation alter the retrograde signal that is sent from RYR1 to the DHPR, delaying the inactivation of the DHPR voltage sensor
Giulivi C, et al. [172] 2011	R163C	Basal bioenergetic abnormalities in skeletal muscle from ryanodine receptor malignant hyperthermia-susceptible R163C knock-in mice	Chronically elevated resting Ca^2+^ in R163C skeletal muscle elicited the maintenance of a fast-twitch fiber program and the development of insulin resistance-like phenotype as part of a metabolic adaptation to the R163C RyR1 mutation
Feng W, et al. [173] 2011	R163C	Functional and biochemical properties of ryanodine receptor type 1 channels from heterozygous R163C malignant hyperthermia-susceptible mice	R163C channels are inherently more active than WT channels, a functional impairment that cannot be reversed by dephosphorylation with protein phosphatase. Dysregulated R163C channels produce a more overt phenotype in myotubes than in adult fibers in the absence of triggering agents, suggesting tighter negative regulation of R163C-RyR1 within the Ca^2+^ release unit of adult fibers
Estève E, et al. [174] 2010	R163C	A malignant hyperthermia-inducing mutation in RYR1 (R163C): alterations in Ca^2+^ entry, release, and retrograde signaling to the DHPR	Conformational changes induced by the R163C MH mutation alter the retrograde signal that is sent from RYR1 to the DHPR, delaying the inactivation of the DHPR voltage sensor and enhancing sarcolemmal Ca^2+^ entry during depolarization
Bannister RA, et al. [175] 2010	R163C	A malignant hyperthermia-inducing mutation in RYR1 (R163C): consequent alterations in the functional properties of DHPR channels	Mutations in RYR1 can alter DHPR activity and raise the possibility that this altered DHPR function may contribute to MH episodes
Cherednichenko G, et al. [176] 2008	R163C	Enhanced excitation-coupled calcium entry in myotubes expressing malignant hyperthermia mutation R163C is attenuated by dantrolene	Myotubes isolated from mice heterozygous and homozygous for the ryanodine receptor type 1 R163C MH susceptibility mutation show significantly enhanced ECCE rates that could be restored to those measured in wild-type cells after exposure to clinical concentrations of dantrolene
Yang T, et al. [177] 2006	R163C	Pharmacologic and functional characterization of malignant hyperthermia in the R163C RyR1 knock-in mouse	The newly developed R163C Het mouse line is a valid animal model for studying the largely unknown pathophysiology of MH
Other rodent models			
Brennan S, et al. [178] 2019	T4706M/Indel (equivalent to T4709M in humans)	Mouse model of severe recessive RYR1-related myopathy	The first mouse model of severe, early-onset recessive *RYR1*-RM Mice exhibit clearly observable, early-onset phenotypes, premature mortality and a consistent pattern of myofibre hypotrophy
Elbaz M, et al. [179] 2019	Q1970fsX16/A4329D (equivalent in humans)	Quantitative RyR1 reduction and loss of calcium sensitivity of RyR1Q1970fsX16+ A4329D cause cores and loss of muscle strength	The phenotype of the RyR1Q1970fsX16 + A4329D compound heterozygous mice recapitulates the clinical picture of multiminicore patients and provide evidence of the molecular mechanisms responsible for skeletal muscle defects
Elbaz M, et al. [180] 2019	Q1970fsX16 (equivalent in humans)	Quantitative reduction of RyR1 protein caused by a single-allele frameshift mutation in RYR1 ex36 impairs the strength of adult skeletal muscle fibres	The RyR1Q1970fsX16 mouse model provides mechanistic insight concerning the phenotype of the parent carrying the RYR1 exon 36 mutation and suggests that in skeletal muscle fibres there is a functional reserve of RyR1
RYR-1 Foundation [181] 2019	T4706M/S1669C + L1716 del	Unpublished - https://wwwryr1org/mice	Phenotype includes kyphosis and malocclusion. The model is still being fully characterized
Dulhunty AF, et al. [182] 2019	P3528S	Unpublished - https://wwwryr1org/edamame	Phenotype includes mild scoliosis and decreased mobility (heterozygous) and scoliosis, decreased mobility, hang time, and increased calcium sensitivity. The model is still being fully characterized
Lopez JR, et al. [183] 2018	G2435R	Malignant hyperthermia, environmental heat stress, and intracellular calcium dysregulation in a mouse model expressing the pG2435R variant of RYR1	RYR1 G2435R mice demonstrated gene dose-dependent in vitro and in vivo responses to pharmacological and environmental stressors that parallel those seen in patients with the human RYR1 variant G2434R
Hernandez-Ochoa EO, et al. [184] 2018	L3625D	Loss of S100A1 expression leads to Ca^2+^ release potentiation in mutant mice with disrupted CaM and S100A1 binding to CaMBD2 of RyR1	RyR1D-S100A1KO muscle fibers exhibit a modest but significant increase in myoplasmic Ca^2+^ transients and enhanced Ca^2+^ release flux following field stimulation when compared to fibers from RyR1D mice
Bannister RA, et al. [185] 2016	E4242G	Distinct Components of Retrograde Ca(V)11-RyR1 Coupling Revealed by a Lethal Mutation in RyR1	E4242G markedly reduces L-type current density, CaV11 Po, and CaV11 expression, where this last effect is most likely a consequence of the absence of EC coupling. The effects of E4242G on current density, relative Po, and channel expression are similar to those occurring in dyspedic myotubes
Hanson MG, et al. [186] 2016	E4242G	Potassium dependent rescue of a myopathy with core-like structures in mouse	Amelioration of potassium leaks through potassium homeostasis mechanisms may minimize muscle damage of myopathies due to certain *RYR1* mutations
Hanson MG, et al. [187] 2015	E4242G	Rectification of muscle and nerve deficits in paralyzed ryanodine receptor type 1 mutant embryos	Contractility can be resumed through the masking of a potassium leak, and evoked vesicular release can be resumed via bypassing the defect in RyR1 induced calcium release
Yuen B, et al. [188] 2012	T4826I	Mice expressing T4826I-RYR1 are viable but exhibit sex- and genotype-dependent susceptibility to malignant hyperthermia and muscle damage	T4826I mice underscore the importance of gene × environment interactions in expression of clinical and subclinical phenotype, and suggest that individuals with RyR1 mutations may represent particularly vulnerable populations to environmental stressors
Barrientos GC, et al. [189] 2012	T4826I	Gene dose influences cellular and calcium channel dysregulation in heterozygous and homozygous T4826I-RYR1 malignant hyperthermia-susceptible muscle	Pronounced abnormalities inherent in T4826I-RYR1 channels confer MHS and promote basal disturbances of excitation-contraction coupling, [Ca^2+^](rest), and oxygen consumption rates. Considering that both Het and Hom T4826I-RYR1 mice are viable, the remarkable isolated single channel dysfunction mediated through this mutation in S4-S5 cytoplasmic linker must be highly regulated in vivo
Andersson DC, et al. [190] 2012	S2844A	Stress-induced increase in skeletal muscle force requires protein kinase A phosphorylation of the ryanodine receptor	The molecular mechanism underlying skeletal muscle inotropy requires enhanced SR Ca^2+^ release due to PKA phosphorylation of S2844 in RyR1
Andersson DC, et al. [191] 2011	S2844D	Ryanodine receptor oxidation causes intracellular calcium leak and muscle weakness in aging	6-month-old mice harboring leaky S2844D mutant channels exhibited skeletal muscle defects comparable to 24-month-old WT mice
Yamaguchi N, et al. [192] 2011	L3625D	Modulation of sarcoplasmic reticulum Ca^2+^ release in skeletal muscle expressing ryanodine receptor impaired in regulation by calmodulin and S100A1	L3625D removes both an early activating effect of S100A1 and CaM and delayed suppressing effect of CaCaM on RyR1 Ca^2+^ release
Felder E, et al. [193] 2002	RYR1/DHPR double KO	Morphology and molecular composition of sarcoplasmic reticulum surface junctions in the absence of DHPR and RyR in mouse skeletal muscle	RyR nor DHPR, alone or separately, are necessary for T-SR docking and for the targeting and/or association of calsequestrin and triadin in the junctional SR. Both proteins are needed for appropriate muscle development

#### Other animal model systems

The pathomechanism, diagnosis, and acute treatment of malignant hyperthermia was investigated in 24 publications that used the R615C porcine model system [27, 239–261], Table 6. A number of other preclinical model systems have been described including avian, zebrafish, *C. elegans*, canine, equine, and drosophila, Table 7. Six publications reported on a single recessive zebrafish model system of *RYR1*-RM termed the relatively relaxed (ryr^mi340^) mutant [28, 263–267] which was utilized for high-throughput drug screening [263] and testing of *N*-acetylcysteine as a potential therapeutic to address elevated oxidative stress [264]. A further six publications reported using *Caenorhabditis elegans* (*C. elegans*) with variants in *unc68*, the *RYR1* ortholog [32, 280–284]. With 40% sequence homology to humans, *C. elegans* have been used to investigate RyR1 functional sites [281] and test the potential impact of *RYR1* mutations on central nervous system function [32]. A single heterozygous canine model system of malignant hyperthermia was reported. The canine model system carried a single missense substitution, V547A, affecting the RyR1 cytosolic shell domain and was characterized by responsiveness to an in vivo halothane-succinylcholine challenge and having a positive in vitro contracture test [278]. Four publications described equine model systems of malignant hyperthermia and exertional rhabdomyolysis that carried variations in the *RYR1* gene [274–277]. Two *RYR1* variants were reported: (a) R2454G associated with fulfilment malignant hyperthermia and a high affinity for ryanodine binding [277] and (b) C7360G associated with both anesthetic-induced malignant hyperthermia and exertional/non-exertional rhabdomyolysis [276]. Three publications reported on drosophila with variations in the equivalent *RYR1* gene (dRyr) [271–273]. A total of nine *RYR1* variations were presented comprising eight missense substitutions and one insertion, Table 7. Missense substitutions in drosophila dRyr conferred halothane sensitivity [272], and drosophila with CRISPR/Cas9 gene-edited dRyr have been used to investigate insecticide resistance [271]. Three publications utilized transfected wild-type rodent cells to generate *RYR1* model systems with clinically-relevant variations [268–270] and four reported on the avian crooked neck dwarf mutant which lacks the alpha RyR isoform homologous to human RyR1 [285–288].

**Table 6:** Porcine *RYR1* model system of malignant hyperthermia

Author/Year	Genotype(s)	Title	Conclusions
Popovski ZT, et al. [239] 2016	R615C	Associations of Biochemical Changes and Maternal Traits with Mutation 1843 (C > T) in the *RYR1* Gene as a Common Cause for Porcine Stress Syndrome	Stress susceptible animals have an increased number of stillborn piglets and a reduced number of newborn piglets compared with heterozygous and normal animals
Scheffler TL, et al. [240] 2014	R615C	Fiber hypertrophy and increased oxidative capacity can occur simultaneously in pig glycolytic skeletal muscle	RyR1 R615C increased mitochondrial proteins and DNA, but this was not associated with improved oxidative capacity, suggesting that altered energy metabolism in RyR1 R615C muscle influences mitochondrial proliferation and protein turnover
Bina S, et al. [241] 2010	R615C	Lymphocyte-based determination of susceptibility to malignant hyperthermia: a pilot study in swine	4CmC stimulation of porcine lymphocytes induces increased adenosine formation in MHS cells relative to those from normal swine
Liang X, et al. [242] 2009	R615C	Impaired interaction between skeletal ryanodine receptors in malignant hyperthermia	Purified RyR1(R615C) from MH susceptible porcine skeletal muscle shows significantly reduced oligomerization when compared to RyR1(WT), indicating a potential loss of intrinsic intermolecular control
Ta TA, et al. [243] 2007	R615C	Ryanodine receptor type 1 (RyR1) possessing malignant hyperthermia mutation R615C exhibits heightened sensitivity to dysregulation by non-coplanar 2,2′,3,5′,6-pentachlorobiphenyl (PCB 95)	A genetic mutation known to confer susceptibility to pharmacological agents also enhances sensitivity to an environmental contaminant
Stinckens A, et al. [244] 2007	R615C	The RYR1 g.1843C > T mutation is associated with the effect of the IGF2 intron3-g.3072G > A mutation on muscle hypertrophy	The effect of IGF2 on muscle growth might partially be mediated by the calpain/calpastatin system and that this is dependent on RYR1-mediated Ca^2+^ transport
Murayama T, et al. [245] 2007	R615C	Postulated role of interdomain interaction between regions 1 and 2 within type 1 ryanodine receptor in the pathogenesis of porcine malignant hyperthermia	Stimulation of the RyR1MHS channel caused by affected inter-domain interaction between regions 1 and 2 is an underlying mechanism for dysfunction of Ca^2+^ homoeostasis seen in the MH phenotype
McKinney LC, et al. [262] 2006	R615C	Characterization of Ryanodine Receptor–mediated Calcium Release in Human B Cells	Lymphocytes from MH pigs displayed an increased sensitivity to 4-CmC (EC50 decreased from 0.81 mM to 0.47 mM). The twofold magnitude of the shift was similar to that observed for 4-CmC–sensitive H-ryanodine binding in MH porcine skeletal muscle
Gallant EM, et al. [246] 2004	R615C	Caffeine sensitivity of native RyR channels from normal and malignant hyperthermic pigs: effects of a DHPR II–III loop peptide	In MH-susceptible pig muscles the caffeine sensitivity of native RyRs was enhanced, the sensitivity of RyRs to a skeletal II–III loop peptide was depressed, and an interaction between the caffeine and peptide CS activation mechanisms seen in normal RyRs was lost
Zhao F, et al. [247] 2001	R615C	Dantrolene inhibition of ryanodine receptor Ca^2+^ release channels. Molecular mechanism and isoform selectivity	Both the RyR1 and the RyR3, but not the RyR2, may be targets for dantrolene inhibition in vivo
Gallant EM, et al. [248] 2001	R615C	Arg(615) Cys substitution in pig skeletal ryanodine receptors increases activation of single channels by a segment of the skeletal DHPR II-III loop	Enhanced DHPR activation of RyRs may contribute to increased Ca^2+^ release from SR in MH-susceptible muscle
Balog EM, et al. [249] 2001	R615C	Divergent effects of the malignant hyperthermia-susceptible Arg(615)-- > Cys mutation on the Ca^2+^ and Mg^2+^ dependence of the RyR1	Reduced Mg^2+^ inhibition of the MHS RyR1 compared with the normal RyR1 is due to both an enhanced selectivity of the MHS RyR1 A-site for Ca^2+^ over Mg^2+^ and a reduced Mg^2+^ affinity of the I-site
Dietze B, et al. [250] 2000	R615C	Malignant hyperthermia mutation Arg615Cys in the porcine ryanodine receptor alters voltage dependence of Ca^2+^ release	Arg615Cys does not only promote ligand-induced Ca^2+^ release but also the depolarization-induced release controlled by the DHP receptor voltage sensor
Laver DR, et al. [251] 1997	R615C	Reduced inhibitory effect of Mg^2+^ on ryanodine receptor-Ca2+ release channels in malignant hyperthermia	The cytoplasmic Mg^2+^ in vivo (approximately 1 mM), this Ca^2+^ Mg^2+^ inhibitory site will be close to fully saturated with Mg^2+^ in normal RyRs, but less fully saturated in MHS RyRs. Therefore, MHS RyRs should be more sensitive to any activating stimulus, which would readily account for the development of an MH episode
Fruen BR, et al. [252] 1997	R615C	Dantrolene inhibition of sarcoplasmic reticulum Ca^2+^ release by direct and specific action at skeletal muscle ryanodine receptors	Results demonstrate selective effects of dantrolene on skeletal muscle ryanodine receptors that are consistent with the actions of dantrolene in vivo and suggest a mechanism of action in which dantrolene may act directly at the skeletal muscle ryanodine receptor complex to limit its activation by calmodulin and C^2+^
Bašić I, et al. [253] 1997	R615C	Stress syndrome: Ryanodine receptor (RYR1) gene in malignant hyperthermia in humans and pigs	This study confirmed application of a method for large-scale, rapid, accurate, DNA-based laboratory diagnosis of the mutation associated with susceptibility to porcine stress syndrome
O’Driscoll S, et al. [254] 1996	R615C	Calmodulin sensitivity of the sarcoplasmic reticulum ryanodine receptor from normal and malignant-hyperthermia-susceptible muscle	The central region of RYR1 is a potential binding domain for CaM in the absence of Ca^2+^. It is suggested that in vivo an enhanced CaM sensitivity of RYR1 might contribute to the abnormal high release of Ca^2+^ from the SR of MHS muscle
Herrmann-Frank A, et al. [255] 1994	R615C	4-Chloro-m-cresol: a specific tool to distinguish between malignant hyperthermia-susceptible and normal muscle	4-CmC is suggested to be a potent tool to distinguish between Ca^2+^ release from MHS and normal muscle
Vogeli P, et al. [256] 1994	R615C	Co-segregation of the malignant hyperthermia and the Arg615-Cys615 mutation in the skeletal muscle calcium release channel protein in five European Landrace and Pietrain pig breeds	DNA-based detection of the MH status in 238 MH-susceptible heterozygous (N/n) and homozygous (n/n) pigs was shown to be accurate, eliminating the 2% diagnostic error that is associated with the halothane challenge test
Ledbetter MW, et al. [257] 1994	R615C	Tissue distribution of ryanodine receptor isoforms and alleles determined by reverse transcription polymerase chain reaction	The normal (Arg615) and mutant (Cys615) ryr1 alleles were expressed in the brains of normal and malignant hyperthermia susceptible pigs, respectively. These results thus demonstrate expression of two ryr isoforms in each type of striated muscle, and all ryr isoforms in a number of regions of the nervous system. The wide distribution of ryr1 in the brain provides a possible neurogenic etiology of malignant hyperthermia
Fagerlund T, et al. [258] 1994	R615C	Search for three known mutations in the *RYR1* gene in 48 Danish families with malignant hyperthermia	Other mutations must underlie the disorder in most Danish malignant hyperthermia-susceptible families, and the “pig mutation” is not a frequent cause of malignant hyperthermia susceptibility in Denmark
Otsu K, et al. [259] 1992	R615C	Refinement of diagnostic assays for a probable causal mutation for porcine and human malignant hyperthermia	PCR-amplified sequences contain constant internal controls for the reliable differentiation by restriction endonuclease digestion of normal, heterozygous, and MH genotypes
Hogan K, et al. [260] 1992	R615C	A cysteine-for-arginine substitution (R614C) in the human skeletal muscle calcium release channel cosegregates with malignant hyperthermia	The cysteine-for-arginine mutation represents a shared calcium release channel pathogenesis between porcine malignant hyperthermia and a subset of mutations responsible for the human malignant hyperthermia syndrome
Otsu K, et al. [261] 1991	R615C	Cosegregation of porcine malignant hyperthermia and a probable causal mutation in the skeletal muscle ryanodine receptor gene in backcross families	Substitution of T for C at nucleotide 1843 is the causative mutation in porcine MH
Fujii J, et al. [27] 1991	R615C	Identification of a mutation in porcine ryanodine receptor associated with malignant hyperthermia	A single point mutation in the porcine gene for the skeletal muscle ryanodine receptor (ryr1) was found to be correlated with MH in five major breeds of lean, heavily muscled swine

**Table 7:** Other *RYR1* preclinical model systems

Author/Year	Species/***RYR1*** variant(s)	Title	Conclusions
Gupta VA, et al. [263] 2013	Zebrafish (Danio rerio)/ ryr1b mi340	Developing therapies for congenital myopathies by high throughput chemical screening in ryanodine receptor 1 mutant zebrafish	A secondary screen using individual chemicals from positive pools is in progress to identify the best combination of chemical/s that improve muscle function and survival of ryr1b mutant fish
Dowling JJ, et al. [28] 2012	Zebrafish (Danio rerio)/ ryr1b mi340	Oxidative stress and successful antioxidant treatment in models of RYR1-related myopathy	Oxidative stress is an important pathophysiological mechanism in *RYR1*-related myopathies and that *N*-acetylcysteine is a successful treatment modality ex vivo and in a vertebrate disease model
Dowling JJ, et al. [264] 2011	Zebrafish (Danio rerio)/ ryr1b mi340	Increased oxidative stress and successful antioxidant treatment in a vertebrate model of RYR1 related myopathy	Increased oxidative stress is an important aspect of the pathogenesis of RYR1-related myopathies, and antioxidant treatment is a viable potential treatment strategy for patients
Dowling JJ, et al. [265] 2010	Zebrafish (Danio rerio)/ ryr1b mi340	Oxidative stress and RYR1-related myopathies	Loss of *RYR1* function in the zebrafish results in increased levels of basal oxidative stress and increased susceptibility to pro-oxidants. *RYR1* deficient zebrafish treated with anti-oxidants had significant improvements in motor function
Dowling JJ, et al. [266] 2009	Zebrafish (Danio rerio)/ ryr1b mi340	Oxidative stress and antioxidant therapy in a zebrafish model of multi minicore myopathy	Loss of *RYR1* function results in increased oxidative stress in a vertebrate model of *RYR1* related myopathy. Antioxidant therapy can improve motor function
Hirata H, et al. [267] 2007	Zebrafish (Danio rerio)/ ryr1b mi340	Zebrafish relatively relaxed mutants have a ryanodine receptor defect, show slow swimming and provide a model of multi-minicore disease	Zebrafish relatively relaxed mutants may be useful for understanding the development and physiology of MmD
Oyamada H, et al. [268] 2002	Hamster (CHO cells)/ P4667S, L4837V, R615C	Novel mutations in C-terminal channel region of the ryanodine receptor in malignant hyperthermia patients	L4838V was responsible for increased sensitivity of RyR1 to caffeine P4667S had very little effect on the caffeine-induced Ca^2+^ increase
Treves S, et al. [84] 1994	Monkey (COS-7 cells)/ R615C	Alteration of intracellular Ca^2+^ transients in COS-7 cells transfected with the cDNA encoding skeletal-muscle ryanodine receptor carrying a mutation associated with malignant hyperthermia.	Presence of the Arg-to-Cys point mutation in the recombinant RYR expressed in COS-7 transfected cells causes abnormal cytosolic Ca^2+^ transients in response to 4-chloro-m-cresol, an agent capable of eliciting in vitro contracture of MH-susceptible muscles.
Altafaj X, et al. [85] 2005	Monkey (COS-7 cells)/ RyR1ΔF7 (3241-3661Del)	Maurocalcine and domain A of the II-III loop of the dihydropyridine receptor Cav 1.1 subunit share common binding sites on the skeletal ryanodine receptor.	RyR1 carrying a deletion of fragment 7 shows a loss of interaction with both peptide A and maurocalcine. This deletion abolishes the maurocalcine induced stimulation of [3H] ryanodine binding onto microsomes of transfected COS-7 cells without affecting the caffeine and ATP responses.
Vega AV, et al. [269] 2011	C2C12 (mouse)/ Y524S, I4897T	Calcitonin gene-related peptide restores disrupted excitation-contraction coupling in myotubes expressing central core disease mutations in RyR1	Changes in excitation–contraction coupling induced by the expression of RyR1 channels bearing CCD mutations Y523S or I4897T can be reversed by calcitonin gene related peptide
Lefebvre R, et al. [270] 2011	Swiss OF1 (mouse)/Y523S, R615C, R2163H, I4897T	Defects in Ca2+ release associated with local expression of pathological ryanodine receptors in mouse muscle fibres	The Y523S, R615C and R2163H RyR1 mutants produce a similar over-sensitive activation of the calcium flux whereas I4897T RyR1 mutants are responsible for a depressed Ca^2+^ flux. The alterations appear to result from inherent modifications of RyR1 channel function and not from indirect changes in the muscle fibre homeostasis
Douris V, et al. [271] 2017	Drosophila model/G4946V, G4946E, I4790M	Investigation of the contribution of RyR target-site mutations in diamide resistance by CRISPR/Cas9 genome modification in Drosophila	Mutations confer subtle differences on the relative binding affinities of the three diamides at an overlapping binding site on the RyR protein
Gao S, et al. [272] 2013	Drosophila model/ Q3878X, Y4452X, R4305C, E4340K, P2773L	Drosophila ryanodine receptors mediate general anesthesia by halothane	Neurally expressed dRyr mediates a substantial proportion of the anesthetic effects of halothane in vivo, is potently activated by halothane in vitro, and activates an inhibitory conductance
Sullivan KM, et al. [273] 2000	Drosophila model/Ryr16ins	The ryanodine receptor is essential for larval development in *Drosophila melanogaster*	The ryanodine receptor is required for proper muscle function and may be essential for excitation-contraction coupling in larval body wall muscles Results do not support a role for Ryr in normal light responses
Wilberger MS, et al. [274] 2015	Equine model/ C7360G	Prevalence of exertional rhabdomyolysis in endurance horses in the Pacific Northwestern United States	Exertional rhabdomyolysis in this group was not associated with known genetic mutations tied to type 1 PSSM and MH
Nieto JE, et al. [275] 2009	Equine model/ C7360G	A rapid detection method for the ryanodine receptor 1 (C7360G) mutation in Quarter Horses	Genotyping by melting curve analysis with hybridization probes is a rapid and accurate detection method for the RyR1 C7360G mutation that works on both cDNA and gDNA
Aleman M, et al. [276] 2009	Equine model/ C7360G	Malignant hyperthermia associated with ryanodine receptor 1 (C7360G) mutation in Quarter Horses	MH is a potentially fatal disease of Quarter Horses that could be triggered by halogenated anesthetics and other nonanesthetic factors that may include exercise, stress, breeding, illnesses, and concurrent myopathies
Aleman M, et al. [277] 2004	Equine model/ R2454G	Association of a mutation in the ryanodine receptor 1 gene with equine malignant hyperthermia	A missense mutation in RyR1 is associated with MH in the horse, providing a screening test for susceptible individuals. Ryanodine-binding analysis suggests that long-lasting changes in RyR1 conformation persists in vitro after the triggering event
Roberts MC, et al. [278] 2001	Canine model/V547A	Autosomal dominant canine malignant hyperthermia is caused by a mutation in the gene encoding the skeletal muscle calcium release channel (RYR1)	Autosomal dominant canine MH is caused by a mutation in the gene encoding the skeletal muscle calcium release channel The MHS trait in this pedigree of mixed-breed dogs is in perfect co-segregation with the RYR1 V547A mutation
Baines KN, et al. [279] 2017	*Caenorhabditis elegans*/G341R, R2163H, R2454H, R2458H, R4861H, A4940T, R163C, K3452Q	Aging Effects of Caenorhabditis elegans Ryanodine Receptor Variants Corresponding to Human Myopathic Mutations	Single amino acid modifications in *C. elegans* also conferred a reduction in lifespan and an accelerated decline in muscle integrity with age, supporting the significance of ryanodine receptor function for human aging
Baines KN, et al. [280] 2014	Caenorhabditis elegans (unc-68)/ G341R, R2163H, R2454H, R2458H, R4861H, A4940T, R163C, K3452Q	Caenorhabditis elegans as a model organism for RYR1 variants and muscle ageing	The ryanodine receptor in Caenorhabditis elegans is UNC-68, which has 40% amino acid identity to the human protein
Hamada T, et al. [281] 2002	Caenorhabditis elegans/ *kh30, e540, × 14, r1161*	Molecular dissection, tissue localization and Ca^2+^ binding of the ryanodine receptor of Caenorhabditis elegans	We propose a model for the functional domains of CeRyR, which agrees well with the model of mammalian skeletal RyR, which is based on proteolysis and cross-linking analysis
Maryon EB, et al. [282] 1998	Caenorhabditis elegans (unc-68)/ *r1161, r1162, r1221, e540*	Muscle-specific functions of ryanodine receptor channels in Caenorhabditis elegans	Unlike vertebrates, which have at least three ryanodine receptor genes, C elegans has a single gene encoded by the unc-68 locus. Unc-68 is expressed in most muscle cells, and the phenotypic defects exhibited by unc-68 null mutants result from the loss of unc-68 function in pharyngeal and body-wall muscle cells
Sakube Y, et al. [283] 1997	Caenorhabditis elegans (unc-68)/ *e540,* unc-68 null	An abnormal ketamine response in mutants defective in the ryanodine receptor gene ryr-1 (unc-68) of Caenorhabditis elegans	S1444N substitution is at a putative protein kinase C phosphorylation site in ryr-1 unc-68(e540) contains a splice acceptor mutation that creates a premature stop codon in the ryr-1 gene
Maryon EB, et al. [284] 1996	Caenorhabditis elegans (unc-68)/ *rl l51, rl152, rl158, rl160, rl161, r1162, r1167, r1207, r1208, rl209, r1210, r1211, r1212, rDfl, rDf2*	unc-68 encodes a ryanodine receptor involved in regulating C elegans body-wall muscle contraction	The role of RyRs in C elegans body-wall muscle is to enhance contraction by amplifying a depolarization-coupled Ca^2+^ transient
Airey JA, et al. [285] 1993	Crooked Neck Dwarf (cn/cn) alpha RyR-null	Failure to make normal alpha ryanodine receptor is an early event associated with the crooked neck dwarf (cn) mutation in chicken	Failure to make normal alpha RyR receptor appears to be an event closely associated with the cn mutation and one which may be largely responsible for development of the cn/cn phenotype in embryonic skeletal muscle
Airey JA, et al. [286] 1993	Crooked Neck Dwarf (cn/cn) alpha RyR-null	Crooked neck dwarf (cn) mutant chicken skeletal muscle cells in low density primary cultures fail to express normal alpha ryanodine receptor and exhibit a partial mutant phenotype	The mutant phenotype observed in ovo is partially expressed under low density culture conditions, and neither beta RyR protein nor its function appear to be capable of preventing the associated changes
Ivanenko A, et al. [287] 1995	Crooked Neck Dwarf (cn/cn) alpha RyR-null	Embryonic chicken skeletal muscle cells fail to develop normal excitation-contraction coupling in the absence of the alpha ryanodine receptor. Implications for a two-ryanodine receptor system	In the absence of alpha RyR there is a failure to develop Ca^2+^ − independent Ca^2+^ release and contractions and to sustain Ca^2+^ − dependent release. Moreover, contributions by the alpha RyR cannot be duplicated by the beta RyR alone
Oppenheim RW, et al. [288] 1997	Crooked Neck Dwarf (cn/cn) alpha RyR-null	Neuromuscular development in the avian paralytic mutant crooked neck dwarf (cn/cn): further evidence for the role of neuromuscular activity in motoneuron survival	It seems likely that the peripheral excitation of muscle by motoneurons during normal development is a major factor in regulating retrograde muscle-derived (or muscle-associated) signals that control motoneuron differentiation and survival

## Discussion

This comprehensive scoping review of MH and *RYR1*-RM preclinical model systems identified 262 relevant published records and serves as a compendium to guide future research. During the period spanning January 1, 1990 to July 3, 2019 a diverse range of preclinical model systems were utilized to investigate the etiology, pathomechanisms, and potential treatments for MH and *RYR1*-RM. There has been sustained research output since 2010 with the predominant model system used varying over time between porcine, cellular, and rodent.

A single missense substitution, R615C, was the sole porcine variant reported. As the first *RYR1* preclinical model system, studies of R615C pigs led to fundamental discoveries including identification of 4-CmC as a potent RyR1 agonist and identification of *RYR1* as a genetic locus for malignant hyperthermia [27, 255]. The R615C porcine model system was also utilized to better understand the mechanism of dantrolene which remains the only approved treatment for MH crises [252].

The number of *RYR1* variations reported in the literature (> 700) has been prohibitive in terms of developing in vivo model systems reflecting each variant. This review has outlined the extent to which cellular model systems, in particular transfected HEK-293 cells and dyspedic myotubes, have been versatile systems through which to investigate the pathogenicity of *RYR1* variations and their impact on intracellular calcium homeostasis. However activity of the RyR1 protein complex is tightly regulated by coupling to the dihydropyridine receptor and by modulators of channel function such as 12-kDa FK506-binding protein (FKBP12) and calmodulin [10]. Absence of these components in the HEK-293 system may therefore affect the reliability of functional data for clinical translation. Epstein-Barr virus-driven immortalization of patient-derived lymphoblasts has also proven a valuable non-recombinant methodology when clinical biospecimens are available, although they also do not contain all elements of the skeletal muscle triad. Both HEK-293 cells and dyspedic myotubes have a standardized and well-characterized background and are therefore less likely, than immortalized patient cells, to be influenced by variations in other genes that may impact RyR1 function. Although patient tissue is not always readily available, it is important to recognize that functional studies of patient-derived primary myotubes can provide valuable supporting evidence of *RYR1* variant pathogenicity. Indeed, such studies have been incorporated within the MH variant scoring matrix developed by the European Malignant Hyperthermia Group (EMHG) [289]. Despite the above limitations, recent advances in the engineering of skeletal muscle three-dimensional systems using patient-derived induced pluripotent stem cells holds the prospect of providing a physiologically relevant cellular system through which to evaluate and screen potential treatments for skeletal muscle disorders, including *RYR1*-RM [290–292]. Our observation that the most frequently reported variants were localized to MH/CCD hotspot regions is consistent with the initial clinical focus to identify patients with variants in these distinct regions and perform functional characterization [293]. Common functional analyses identified in this review include RyR1 agonist sensitivity (caffeine, 4-CmC), 3[H]-ryanodine binding, halothane and/or isoflurane sensitivity, and intracellular calcium measurements via calcium-sensitive fluorescent dyes such as fluo-4.

The dyspedic mouse was utilized by 47% of publications in the rodent category and its *RYR1*-null myotubes were transfected in 23% of publications in the cellular model category, a testament to importance of the dyspedic mouse for both understanding the fundamental physiology of the ryanodine receptor and as a stable model system to characterize mutant RyR1 channels. Heterozygous knock-in rodent models have formed the basis of in vivo *RYR1*-RM preclinical testing. Y524S, I4895T, and R163C were the most extensively studied knock-in rodent models over the last 30 years. These mice have provided valuable insights into the effects of single missense substitutions on RyR1 dysfunction including channel leak and excitation-contraction uncoupling. Furthermore, these mice have enabled the identification downstream pathologic sequalae in vivo such as elevated oxidative/nitrosative/ER stress and an unfolded protein response. However the abovementioned knock-in mice do not necessarily mirror the phenotypes observed in autosomal dominant patients with equivalent *RYR1* variants (reviewed in detail elsewhere [292]). Two recently published compound heterozygous *RYR1*-RM rodent models recapitulate clinical manifestations observed in recessive *RYR1*-RM patients, including decreased RyR1 protein expression, reduced muscle mass, and progressive muscle weakness [178, 179]. An additional compound heterozygous mouse (T4706M/S1669C + L1716del) included in this review is currently undergoing full characterization [181].

In contrast to rodent model systems, zebrafish are more cost-efficient, have transparent embryos that facilitate visualization of dynamic events, have a shorter lifecycle, have larger hatch sizes, and are easier to maintain [294, 295]. Zebrafish are readily manipulated by chemical approaches because embryos can readily absorb compounds that they are exposed to in solution, therefore allowing for high-throughput chemical screening [296, 297]. A recessive zebrafish model system of *RYR1*-RM, termed the relatively relaxed (ryr^mi340^) mutant, exhibits weak muscle contractions resulting in slow swimming, dramatically decreased Ca^2+^ transients at the t-tubules of fast muscles due to defective E-C coupling, and small amorphous cores detectable by electron microscopy. Despite the abovementioned advantages over rodent model systems, a consideration is that relatively relaxed (ryr1b) zebrafish are homozygous with a truncated RyR1 channel (residual expression = 1–10% of normal RyR1). As such, its genetic defect and pathomechanism do not align with a majority of *RYR1*-RM clinical cases.

Consistent with the findings of this review, over 90% of animals used in research are mice or rats [298]. However, other animal model systems have also been developed and used to study the skeletal muscle ryanodine receptor and the consequences of genetic variations. *C. elegans* and drosophila have been primarily used for genetic and developmental biology studies, whereas the porcine, equine, and canine model systems have focused on understanding and characterizing the etiology of MH in these species. As an alternative to higher order *RYR1*-RM animal model systems, the use of simpler organisms (*C. elegans*, yeast, drosophila) and vertebrates (in particular zebrafish) with sufficient genome sequence homology to humans could be revitalized using more precise genome editing techniques such as prime editing [299]. However, due to evolutionary distance between DNA sequences, results from non-mammalian model systems should undergo further careful validation in mammals such as mice and pigs prior to translation to clinical studies. It is possible that records published in supplementary material may not have been captured by the search strategy used for this review and may be considered a limitation.

Advances in functional genomics coupled with the increase in demand for mice as a primary experimental system are expected to continue driving the need for additional transgenic, gene-edited and combinatorial breeding of different *RYR1*-RM model systems in the near future. Development of conditional targeted animal models (cre/lox, tet, and other similar approaches) can reduce generation and retention of extraneous animals and also allow for the conduct of developmental studies in late-onset myopathy subtypes in this heterogeneous group of disorders. Determining which murine model most closely represents a majority of either dominant or recessive cases of *RYR1*-RM remains an open question. Funding of research utilizing recently developed model systems is essential to translating these promising advances into clinical trials and treatment discoveries.

## Conclusion

Over the past 30 years, there were 262 publications on MH and *RYR1*-RM preclinical model systems featuring more than 200 unique *RYR1* variations tested in a broad range of species. Findings from these studies have set the foundation for therapeutic development for MH and *RYR1*-RM.

## Supplementary information

**Additional file 1.**

## Data Availability

Not applicable.

## Abbreviations

4-CmC: 4-Chloromethcathinone
AICAR: 5-aminoimidazole-4-carboxamide ribonucleoside
ApoCaM: Apocalmodulin
ASGS: Allele-specific gene silencing
*C. elegans*: *Caenorhabditis elegans*
CaCaM: Calcium-bound calmodulin
CaM: Calmodulin
CCD: Central Core Disease
CICR: Calcium-induced calcium release
CNMDU1: Congenital neuromuscular disease with uniform type 1 fiber
COS: CV-1 in Origin with SV40 cell line
DHPR: Dihydropyridine receptor
EBV: Epstein-Barr virus
EC: Excitation-contraction
ECCE: Excitation-coupled calcium entry
EMHG: European Malignant Hyperthermia Group
FDB: Flexor digitorum brevis
FKBP12: 12-kDa FK506-binding protein
HEK: Human embryonic kidney cell line
KO: Knock-out
MH: Malignant hyperthermia
MmD: Multiminicore disease
NO: Nitrous oxide
PRISMA: Preferred Reporting Items for Systematic Reviews and Meta-Analyses
ROS: Reactive oxygen species
RVIS: Residual variance intolerance score
*RYR1*-RM: *RYR1*-related myopathies
SOICR: Store overload-induced calcium release
SR: Sarcoplasmic reticulum
UPR: Unfolded protein response
VGCR: Voltage-gated calcium release
WT: Wild type
