# Supplementary material for: Preclinical model systems of ryanodine receptor 1-related myopathies and malignant hyperthermia: a comprehensive scoping review of works published 1990–2019
Source: Orphanet J Rare Dis. 2020 May 7;15:113. doi: 10.1186/s13023-020-01384-x (PMC7204063; doi:10.1186/s13023-020-01384-x)
Supplement: Supplementary file 1 — Additional file 1. [file 13023_2020_1384_MOESM1_ESM.docx]

**Supplementary material**

Preclinical Models of Ryanodine Receptor 1-Related Myopathies: A Comprehensive Scoping Review of Works Published 1990-2019.

Full search strategy:

Review of published literature describing pre-clinical models (i.e. animal models, cell culture models) of ryanodine receptor-1 – related myopathies/disorders (RYR1-RM).

PubMed Literature Search Strategy

Month Searched: April 3-4, 2019, Update July 3, 2019

RYR-1[tiab] OR RYR1[tiab] OR RyR1s[tiab] OR ryanodine receptor calcium release channel[majr] OR “ryanodine receptor 1”[tiab]

AND

malignant hyperthermia[mh] OR “malignant hyperthermia”[tiab] OR malignant hyperpyrexia*[tiab] OR anesthesia hyperthermia*[tiab]

OR

Muscular diseases[mh] OR muscular diseases*[tiab] OR myopathies[tw] OR myopathy[tw] OR muscle[tw] OR muscular[tw] OR muscle contraction[mh] OR muscle contraction*[tiab] OR smooth muscle*[tiab] OR cardiac muscle*[tiab] OR skeletal muscle*[tiab] OR muscle fiber*[tiab] OR myofibril*[tiab]

OR

Cell culture*[tiab] OR cells, cultured[mh] OR cellular culture*[tiab] OR cell line*[tiab] OR muscle cells[mh] OR myocyte*[tiab] OR “muscle cell”[tiab] OR “muscle cells”[tiab] OR myofibril*[tiab] OR "tissue culture"[tiab] OR "in vitro"[tiab]

OR

("animal experimentation"[MeSH Terms] OR "models, animal"[MeSH Terms] OR "invertebrates"[MeSH Terms] OR "Animals"[Mesh:noexp] OR "animal population groups"[MeSH Terms] OR "chordata"[MeSH Terms:noexp] OR "chordata, nonvertebrate"[MeSH Terms] OR "vertebrates"[MeSH Terms:noexp] OR "amphibians"[MeSH Terms] OR "birds"[MeSH Terms] OR "fishes"[MeSH Terms] OR "reptiles"[MeSH Terms] OR "mammals"[MeSH Terms:noexp] OR "primates"[MeSH Terms:noexp] OR "artiodactyla"[MeSH Terms] OR "carnivora"[MeSH Terms] OR "cetacea"[MeSH Terms] OR "chiroptera"[MeSH Terms] OR "elephants"[MeSH Terms] OR "hyraxes"[MeSH Terms] OR "insectivora"[MeSH Terms] OR "lagomorpha"[MeSH Terms] OR "marsupialia"[MeSH Terms] OR "monotremata"[MeSH Terms] OR "perissodactyla"[MeSH Terms] OR "rodentia"[MeSH Terms] OR "scandentia"[MeSH Terms] OR "sirenia"[MeSH Terms] OR "xenarthra"[MeSH Terms] OR "haplorhini"[MeSH Terms:noexp] OR "strepsirhini"[MeSH Terms] OR "platyrrhini"[MeSH Terms] OR "tarsii"[MeSH Terms] OR "catarrhini"[MeSH Terms:noexp] OR "cercopithecidae"[MeSH Terms] OR "hylobatidae"[MeSH Terms] OR "hominidae"[MeSH Terms:noexp] OR "gorilla gorilla"[MeSH Terms] OR "pan paniscus"[MeSH Terms] OR "pan troglodytes"[MeSH Terms] OR "pongo pygmaeus"[MeSH Terms]) OR ((animals[tiab] OR animal[tiab] OR mice[Tiab] OR mus[Tiab] OR mouse[Tiab] OR murine[Tiab] OR woodmouse[tiab] OR rats[Tiab] OR rat[Tiab] OR murinae[Tiab] OR muridae[Tiab] OR cottonrat[tiab] OR cottonrats[tiab] OR hamster[tiab] OR hamsters[tiab] OR cricetinae[tiab] OR rodentia[Tiab] OR rodent[Tiab] OR rodents[Tiab] OR pigs[Tiab] OR pig[Tiab] OR swine[tiab] OR swines[tiab] OR piglets[tiab] OR piglet[tiab] OR boar[tiab] OR boars[tiab] OR "sus scrofa"[tiab] OR ferrets[tiab] OR ferret[tiab] OR polecat[tiab] OR polecats[tiab] OR "mustela putorius"[tiab] OR "guinea pigs"[Tiab] OR "guinea pig"[Tiab] OR cavia[Tiab] OR callithrix[Tiab] OR marmoset[Tiab] OR marmosets[Tiab] OR cebuella[Tiab] OR hapale[Tiab] OR octodon[Tiab] OR chinchilla[Tiab] OR chinchillas[Tiab] OR gerbillinae[Tiab] OR gerbil[Tiab] OR gerbils[Tiab] OR jird[Tiab] OR jirds[Tiab] OR merione[Tiab] OR meriones[Tiab] OR rabbits[Tiab] OR rabbit[Tiab] OR hares[Tiab] OR hare[Tiab] OR diptera[Tiab] OR flies[Tiab] OR fly[Tiab] OR dipteral[Tiab] OR drosphila[Tiab] OR drosophilidae[Tiab] OR cats[Tiab] OR cat[Tiab] OR carus[Tiab] OR felis[Tiab] OR nematoda[Tiab] OR nematode[Tiab] OR nematoda[Tiab] OR nematode[Tiab] OR nematodes[Tiab] OR sipunculida[Tiab] OR dogs[Tiab] OR dog[Tiab] OR canine[Tiab] OR canines[Tiab] OR canis[Tiab] OR sheep[Tiab] OR sheeps[Tiab] OR mouflon[Tiab] OR mouflons[Tiab] OR ovis[Tiab] OR goats[Tiab] OR goat[Tiab] OR capra[Tiab] OR capras[Tiab] OR rupicapra[Tiab] OR chamois[Tiab] OR haplorhini[Tiab] OR monkey[Tiab] OR monkeys[Tiab] OR anthropoidea[Tiab] OR anthropoids[Tiab] OR saguinus[Tiab] OR tamarin[Tiab] OR tamarins[Tiab] OR leontopithecus[Tiab] OR hominidae[Tiab] OR ape[Tiab] OR apes[Tiab] OR pan[Tiab] OR paniscus[Tiab] OR "pan paniscus"[Tiab] OR bonobo[Tiab] OR bonobos[Tiab] OR troglodytes[Tiab] OR "pan troglodytes"[Tiab] OR gibbon[Tiab] OR gibbons[Tiab] OR siamang[Tiab] OR siamangs[Tiab] OR nomascus[Tiab] OR symphalangus[Tiab] OR chimpanzee[Tiab] OR chimpanzees[Tiab] OR prosimians[Tiab] OR "bush baby"[Tiab] OR prosimian[Tiab] OR bush babies[Tiab] OR galagos[Tiab] OR galago[Tiab] OR pongidae[Tiab] OR gorilla[Tiab] OR ORgorillas[Tiab] OR pongo[Tiab] OR pygmaeus[Tiab] OR "pongo pygmaeus"[Tiab] OR orangutans[Tiab] OR pygmaeus[Tiab] OR lemur[Tiab] OR lemurs[Tiab] OR lemuridae[Tiab] OR horse[Tiab] OR horses[Tiab] OR pongo[Tiab] OR equus[Tiab] OR cow[Tiab] OR calf[Tiab] OR bull[Tiab] OR chicken[Tiab] OR chickens[Tiab] OR gallus[Tiab] OR quail[Tiab] OR bird[Tiab] OR birds[Tiab] OR quails[Tiab] OR poultry[Tiab] OR poultries[Tiab] OR fowl[Tiab] OR fowls[Tiab] OR reptile[Tiab] OR reptilia[Tiab] OR reptiles[Tiab] OR snakes[Tiab] OR snake[Tiab] OR lizard[Tiab] OR lizards[Tiab] OR alligator[Tiab] OR alligators[Tiab] OR crocodile[Tiab] OR crocodiles[Tiab] OR turtle[Tiab] OR turtles[Tiab] OR amphibian[Tiab] OR amphibians[Tiab] OR amphibia[Tiab] OR frog[Tiab] OR frogs[Tiab] OR bombina[Tiab] OR salientia[Tiab] OR toad[Tiab] OR toads[Tiab] OR "epidalea calamita"[Tiab] OR salamander[Tiab] OR salamanders[Tiab] OR eel[Tiab] OR eels[Tiab] OR fish[Tiab] OR fishes[Tiab] OR pisces[Tiab] OR catfish[Tiab] OR catfishes[Tiab] OR siluriformes[Tiab] OR arius[Tiab] OR heteropneustes[Tiab] OR sheatfish[Tiab] OR perch[Tiab] OR perches[Tiab] OR percidae[Tiab] OR perca[Tiab] OR trout[Tiab] OR trouts[Tiab] OR char[Tiab] OR chars[Tiab] OR salvelinus[Tiab] OR "fathead minnow"[Tiab] OR minnow[Tiab] OR cyprinidae[Tiab] OR carps[Tiab] OR carp[Tiab] OR zebrafish[Tiab] OR zebrafishes[Tiab] OR goldfish[Tiab] OR goldfishes[Tiab] OR guppy[Tiab] OR guppies[Tiab] OR chub[Tiab] OR chubs[Tiab] OR tinca[Tiab] OR barbels[Tiab] OR barbus[Tiab] OR pimephales[Tiab] OR promelas[Tiab] OR "poecilia reticulata"[Tiab] OR mullet[Tiab] OR mullets[Tiab] OR seahorse[Tiab] OR seahorses[Tiab] OR mugil curema[Tiab] OR atlantic cod[Tiab] OR shark[Tiab] OR sharks[Tiab] OR catshark[Tiab] OR anguilla[Tiab] OR salmonid[Tiab] OR salmonids[Tiab] OR whitefish[Tiab] OR whitefishes[Tiab] OR salmon[Tiab] OR salmons[Tiab] OR sole[Tiab] OR solea[Tiab] OR "sea lamprey"[Tiab] OR lamprey[Tiab] OR lampreys[Tiab] OR pumpkinseed[Tiab] OR sunfish[Tiab] OR sunfishes[Tiab] OR tilapia[Tiab] OR tilapias[Tiab] OR turbot[Tiab] OR turbots[Tiab] OR flatfish[Tiab] OR flatfishes[Tiab] OR sciuridae[Tiab] OR squirrel[Tiab] OR squirrels[Tiab] OR chipmunk[Tiab] OR chipmunks[Tiab] OR suslik[Tiab] OR susliks[Tiab] OR vole[Tiab] OR voles[Tiab] OR lemming[Tiab] OR lemmings[Tiab] OR muskrat[Tiab] OR muskrats[Tiab] OR lemmus[Tiab] OR otter[Tiab] OR otters[Tiab] OR marten[Tiab] OR martens[Tiab] OR martes[Tiab] OR weasel[Tiab] OR badger[Tiab] OR badgers[Tiab] OR ermine[Tiab] OR mink[Tiab] OR minks[Tiab] OR sable[Tiab] OR sables[Tiab] OR gulo[Tiab] OR gulos[Tiab] OR wolverine[Tiab] OR wolverines[Tiab] OR minks[Tiab] OR mustela[Tiab] OR llama[Tiab] OR llamas[Tiab] OR alpaca[Tiab] OR alpacas[Tiab] OR camelid[Tiab] OR camelids[Tiab] OR guanaco[Tiab] OR guanacos[Tiab] OR chiroptera[Tiab] OR chiropteras[Tiab] OR bat[Tiab] OR bats[Tiab] OR fox[Tiab] OR foxes[Tiab] OR iguana[Tiab] OR iguanas[Tiab] OR xenopus laevis[Tiab] OR parakeet[Tiab] OR parakeets[Tiab] OR parrot[Tiab] OR parrots[Tiab] OR donkey[Tiab] OR donkeys[Tiab] OR mule[Tiab] OR mules[Tiab] OR zebra[Tiab] OR zebras[Tiab] OR shrew[Tiab] OR shrews[Tiab] OR bison[Tiab] OR bisons[Tiab] OR buffalo[Tiab] OR buffaloes[Tiab] OR deer[Tiab] OR deers[Tiab] OR bear[Tiab] OR bears[Tiab] OR panda[Tiab] OR pandas[Tiab] OR "wild hog"[Tiab] OR "wild boar"[Tiab] OR fitchew[Tiab] OR fitch[Tiab] OR beaver[Tiab] OR beavers[Tiab] OR jerboa[Tiab] OR jerboas[Tiab] OR capybara[Tiab] OR capybaras[Tiab]) NOT medline[subset])

EMBASE Search Strategy

Dates Searched: April 3 - 4, 2019, Update July 3, 2019

('ryanodine receptor 1'/exp/mj OR ryr1:ti,ab)

AND

((('musculoskeletal disease'/exp OR 'muscle disease'/exp OR 'myopathy'/exp OR myopathies:ti,ab OR myopathy:ti,ab OR 'muscle disease':ti,ab OR 'muscle diseases':ti,ab OR muscular) AND diseases:ti,ab OR muscle:ti,ab OR muscular:ti,ab OR 'muscle contraction':ti,ab OR muscle) AND contractions:ti,ab OR muscles:ti,ab OR 'muscle'/exp

OR

'malignant hyperthermia':ti,ab OR 'malignant hyperpyrexia':ti,ab OR 'anesthesia hyperthermia':ti,ab OR 'malignant hyperthermia'/exp

OR

'cell culture'/exp OR 'cell line'/exp OR 'muscle cell'/exp OR ‘Cell culture’:ti,ab OR cellular culture:ti,ab OR ‘cell line’:ti,ab OR ‘cell lines’:ti,ab OR ‘muscle cells’:ti,ab OR myocyte:ti,ab OR “muscle cell”:ti,ab OR “muscle cells”:ti,ab OR ‘smooth muscle’:ti,ab OR ‘cardiac muscle’:ti,ab OR ‘skeletal muscle’:ti,ab OR ‘muscle fiber’:ti,ab OR myofibril:ti,ab OR myofibroblast:ti,ab OR 'cell culture'/exp OR 'cell line'/exp OR 'muscle cell'/exp OR 'in vitro study'/exp

OR

'animal model'/exp OR mouse:ti,ab OR porcine:ti,ab OR pig:ti,ab OR rats:ti,ab OR rat:ti,ab OR cow:ti,ab OR cows:ti,ab OR bovine:ti,ab OR zebrafish:ti,ab OR fish:ti,ab OR fishes:ti,ab OR murine:ti,ab OR animal model:ti,ab OR mammalian:ti,ab OR mammal:ti,ab OR primate:ti,ab OR primates:ti,ab OR monkey:ti,ab OR ape:ti,ab OR apes:ti,ab OR gibbon:ti,ab OR chimpanzee:ti,ab OR gorilla:ti,ab OR macaque:ti,ab OR 'animal experiment'/exp/mj
